# Multi-target-directed phenol–triazole ligands as therapeutic agents for Alzheimer's disease†
†Electronic supplementary information (ESI) available. See DOI: 10.1039/c7sc01269a



**DOI:** 10.1039/c7sc01269a

**Published:** 2017-06-05

**Authors:** Michael R. Jones, Emilie Mathieu, Christine Dyrager, Simon Faissner, Zavier Vaillancourt, Kyle J. Korshavn, Mi Hee Lim, Ayyalusamy Ramamoorthy, V. Wee Yong, Shigeki Tsutsui, Peter K. Stys, Tim Storr

**Affiliations:** a Department of Chemistry , Simon Fraser University , V5A1S6 , Burnaby , BC , Canada . Email: tim_storr@sfu.ca; b Department of Clinical Neurosciences , Hotchkiss Brain Institute , Cumming School of Medicine , University of Calgary , Calgary , Canada; c Department of Neurology , St. Josef-Hospital , Ruhr-University , Bochum , Germany; d Department of Chemistry , University of Michigan , Ann Arbor , USA; e Department of Chemistry , Ulsan National Institute of Science and Technology (UNIST) , Ulsan , Korea; f Department of Biophysics , University of Michigan , Ann Arbor , USA

## Abstract

Alzheimer's disease (AD) is a multifactorial disease that is characterized by the formation of intracellular neurofibrillary tangles and extracellular amyloid-β (Aβ) plaque deposits. Increased oxidative stress, metal ion dysregulation, and the formation of toxic Aβ peptide oligomers are all considered to contribute to the etiology of AD. In this work we have developed a series of ligands that are multi-target-directed in order to address several disease properties. 2-(1-(3-Hydroxypropyl)-1*H*-1,2,3-triazol-4-yl)phenol (**POH**), 2-(1-(2-morpholinoethyl)-1*H*-1,2,3-triazol-4-yl)phenol (**PMorph**), and 2-(1-(2-thiomorpholinoethyl)-1*H*-1,2,3-triazol-4-yl)phenol (**PTMorph**) have been synthesized and screened for their antioxidant capacity, Cu-binding affinity, interaction with the Aβ peptide and modulation of Aβ peptide aggregation, and the ability to limit Aβ_1–42_-induced neurotoxicity in human neuronal culture. The synthetic protocol and structural variance incorporated *via* click chemistry, highlights the influence of R-group modification on ligand-Aβ interactions and neuroprotective effects. Overall, this study demonstrates that the phenol–triazole ligand scaffold can target multiple factors associated with AD, thus warranting further therapeutic development.

## Abstract 2

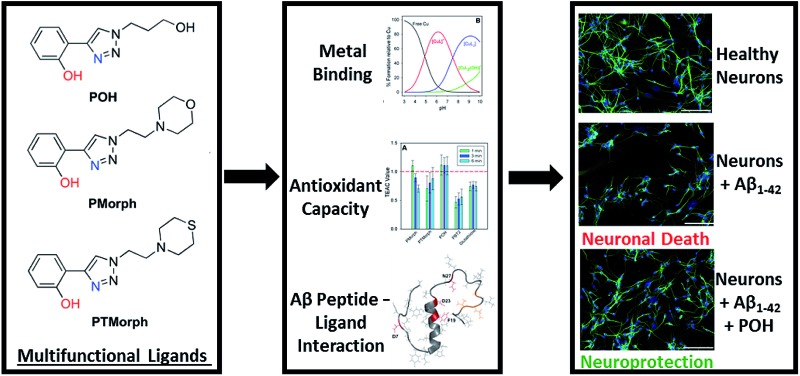
A series of multi-target-directed ligands are described that bind Cu, act as antioxidants, modulate Aβ peptide aggregation, and abolish Aβ toxicity in primary neurons.

## Introduction

Healthcare advances across the globe have increased life expectancy, facilitating increases in the prevalence of neurodegenerative diseases such as Alzheimer's disease (AD).^1,2^ In Canada for example, *ca.* 5% of the population over 65 years of age have AD, increasing to 25% over the age of 85 years old.^3^ Currently, there are no approved disease-modifying therapeutic interventions other than symptomatic treatments such as cholinesterase inhibitors.^4^

AD is formally characterized by two distinct neuropathological features: extracellular amyloid-β (Aβ) plaques and intracellular neurofibrillary tangles (NFTs).^5,6^ The major constituent of Aβ plaques is the Aβ peptide of *ca.* 38–43 amino acid residues, with Aβ_1–40_ and Aβ_1–42_ being the most abundant forms. NFTs result from the hyperphosphorylation of tau proteins. Oxidative stress is linked to the formation of both of these pathological features,^7^ and recent reports suggest an inter-relationship between Aβ and NFTs.^8^

AD is a multifactorial disease where oxidative stress, altered Aβ clearance mechanisms, Aβ and tau aggregation, and dysregulated metal ions play a role in disease etiology.^9^ The N-terminus of the Aβ peptide exhibits a relatively high affinity for Cu (*K*_d_ = 10^–11^ to 10^–7^) and Zn (*K*_d_ = 10^–9^ to 10^–6^) where residues His6, His13, and His14 are located.^10–13^ The concentration of metal ions in Aβ plaque deposits is 3–5 fold higher in comparison to age-matched healthy parenchyma, suggesting that Aβ plaques act as metal reservoirs, as overall metal concentrations in the brain are not altered.^14^ The interaction between metal ions and the Aβ peptide drastically alters the Aβ aggregation profile; interaction with Zn affords amorphous high molecular weight species, while interaction with Cu affords neurotoxic oligomers.^15^ In the case of CuAβ, peptide binding promotes Cu^II^/Cu^I^ redox cycling, and generation of reactive oxygen species (ROS), potentially implicating these metalated species in oxidative stress, and neuronal toxicity.^16–18^

**Fig. 1:**
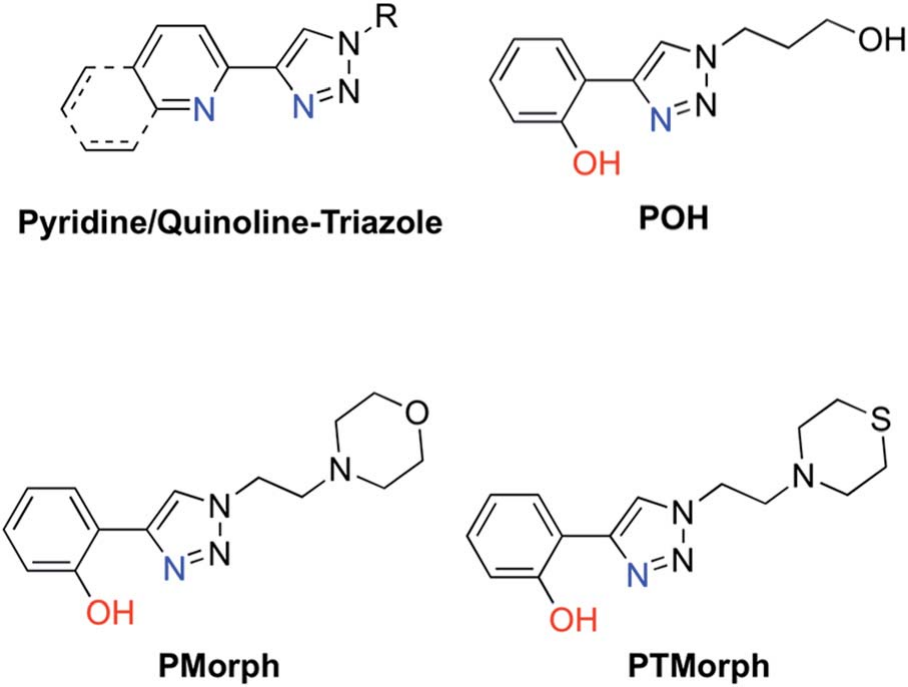
Chemical structures of relevant triazole-containing ligands including **POH**, **PMorph**, and **PTMorph**.

Small molecule chemical agents that can address multiple factors associated with AD etiology may play a key role in the development of new effective therapeutic strategies. Specifically, developing multifunctional agents that can act on multiple disease pathways could provide key information on the interrelationship between these pathways, enhancing our overall understanding of AD etiology. The rational design of multifunctional metal binding agents has become a promising therapeutic strategy.^19–27^ Previously, we developed several multifunctional frameworks based on pyridine–triazole and quinoline–triazole scaffolds (Fig. 1). The pyridine–triazole frameworks were shown to alter metal–Aβ interactions and associated Aβ aggregation.^28^ The quinoline–triazole analogues were developed to provide an enhanced interaction with the hydrophobic region of the Aβ peptide.^29^ In a significant advance, we report herein a series of multi-target directed ligands possessing a phenol–triazole framework (Fig. 1). These ligands exhibit an enhanced affinity for Cu in comparison to the pyridine/quinoline analogues, and in addition, contain an antioxidant phenolic group. The phenol–triazoles were shown to modulate the Aβ aggregation profile in the presence and absence of added Cu based on gel electrophoresis and transmission electron microscopy. 2-D NMR and molecular modelling studies were employed to provide further insight into the ligand–peptide interaction. Finally, one compound in the series (**POH**) was shown to reverse Aβ_1–42_ neurotoxicity in primary neurons.

## Results and discussion

### Ligand properties

AD drug development has targeted several different pathophysiological pathways, including Aβ peptide aggregation, tau protein hyperphosporylation, oxidative stress, cholinesterase inhibition, and metal ion dyshomeostasis.^30^ The lack of a single defined target suggests that an effective therapeutic intervention may require the ability to address several different factors associated with the disease. Herein, a series of phenol–triazoles (**POH**, **PMorph** and **PTMorph**, Fig. 1) were designed to bind Cu, modulate metal–peptide interactions, and limit the generation of reactive oxygen species (ROS) *via* the phenol moiety. The phenol–triazole series were synthesized in a modular fashion using Huisgen's 1,3-dipolar cycloaddition, also known as click chemistry.^31,32^ Reaction of an alkyne substituted phenol and azides with different peripheral R-groups affords a bidentate metal-binding site (Scheme S1†).

We next determined the ligand acidity constants *via* a combination of variable pH UV-Vis and ^1^H NMR spectroscopies (Fig. S1–S3†). The phenol p*K*_a_ values were thus determined to be within the range of 9.54–9.55 (Table S1†), comparable to the deprotonation of free phenol (p*K*_a_ = 9.98 (ref. 33)). The additional p*K*_a_ values for the peripheral morpholine (**PMorph**) and thiomorpholine (**PTMorph**) functions were determined to be 5.5 and 5.6, respectively. These values are lower than free morpholine and thiomorpholine (p*K*_a_ morpholine = 8.36, thiomorpholine = 9.0) as has been previously reported in similar systems.^29,34–36^ Overall, each ligand was found to be neutral at physiological pH 7.4, which is optimal for passive diffusion across the BBB. To further investigate the drug-like properties of the phenol–triazoles, several physicochemical parameters were calculated. Each phenol–triazole ligand was determined to comply with Lipinski's rules and log BB for drug-likeness and BBB penetration (Table S2†).^37,38^ Overall, the phenol–triazole series exhibit promising physicochemical properties warranting further development.

### Metal binding affinity

We next investigated the Cu-binding affinity of the bidentate phenol–triazole ligands in solution using variable pH UV-vis titrations. The Aβ peptide exhibits a high affinity for Cu(ii) (*K*_d_ *ca.* 10^–11^ to 10^–7^)^12,13^ and thus in order to compete with Aβ for Cu, either *via* de-metallation or ternary complex formation, ligands should exhibit a Cu *K*_d_ value in the 10^–12^ to 10^–8^ range.^12^ This range is appropriate to disrupt Cu–Aβ interactions, while limiting sequestration of essential metal ions from metalloproteins.^39^

**Fig. 2:**
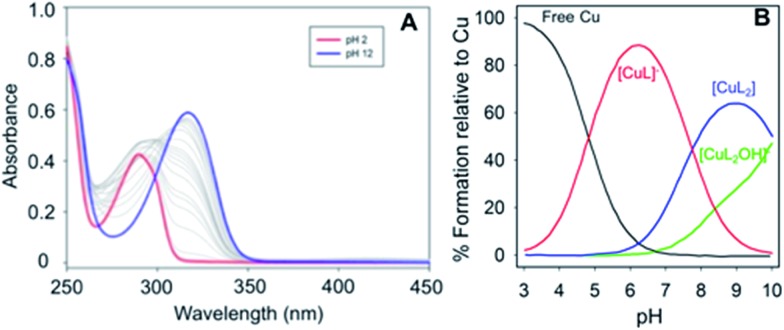
(Left) Variable pH UV-vis titration of **POH** (75 μM) and CuCl_2_ (37.5 μM) where the red spectrum represents pH 2 and the blue spectrum at pH 12. (Right) Using HypSpec and HySS,^43,45^ the variable pH data were fit to a model including a 1 : 1 and 2 : 1 ligand : Cu species along with free Cu and a Cu(**PMorph**)_2_OH component at high pH. At physiological pH 7.4, very little free Cu is present and a combination of 1 : 1 and 2 : 1 ligand : Cu species are present.

The stoichiometry of the Cu(ii) – phenol–triazole complexes in PBS (pH 7.4) was initially determined using Jobs plot analysis (Fig. S4†).^40^ For the example ligand **PMorph**, a broad maxima is observed between *ca.* 0.3–0.6 mole fraction of Cu(ii), suggesting the formation of both 1 : 1 and 1 : 2 **PMorph**–Cu(ii) complexes in solution (*vide infra*).^41,42^ Further, both 1 : 1 and 1 : 2 binding stoichiometries were observed for the three phenol–triazoles by both Jobs plot and ESI-MS analysis (data not shown). Measurement of the binding affinity of the phenol–triazole ligands for Cu(ii) was completed using variable pH spectrophotometric titrations and speciation modelling using HypSpec (Fig. 2).^43^ The ligand p*K*_a_ values, as well as the hydrolysis reactions of free Cu(ii), were included as constants in the calculations.^44^ Modelling of the data for **POH** shows significant log *K* values for both 1 : 1 and 1 : 2 Cu : L ratios (where L is deprotonated) as shown in Table 1. As expected, the calculated log *K* values are similar across the phenol–triazole series (Table 1), reflecting a common metal-binding motif. A representative speciation diagram for **POH** (Fig. 2) shows that at physiological pH 1 : 1 and 1 : 2 Cu : L species predominate (in agreement with Jobs plot analysis) with negligible free Cu. At higher pH, the [CuL_2_(OH)]^–^ species becomes relevant. Similar speciation diagrams for **PMorph** and **PTMorph** are shown in the ESI (Fig. S5 and S6†). Using the solution speciation diagrams, the concentration of unchelated Cu(ii) (pCu = –log([Cu_unchelated_])) at pH 7.4 and total Cu concentration can be calculated (Table 1). The pCu value is a direct estimate of the ligand–Cu affinity by taking into account all relevant equilibria, and can therefore be used to compare the metal-binding affinity among the various ligands, including the Aβ peptide. The pCu values are similar across the phenol–triazole series (6.6–6.9), and represent approximate dissociation constants (nM to μM range) that compare favourably with the *K*_d_ values reported for Cu–Aβ species.^12,13^ The Cu-binding affinity for the phenol–triazole ligands are thus significantly stronger than those reported for the quinoline–triazole analogues.^29^ A Cu-competition assay was then performed in which 2 eq. of each phenol–triazole ligand was added to pre-formed Cu–Aβ_1–16_ and Cu–Aβ_1–42_ species. The Cu complex of the phenol–triazole ligands displays an absorption peak at *ca.* 320 nm (Fig. S7–S9†), and upon addition of ligand to solutions containing Cu–Aβ_1–42/1–16_, an increase at 320 nm is observed. This data suggests an interaction of the phenol–triazole ligands with Cu. A baseline increase in the Aβ_1–42_ experiments is likely due to aggregate formation and associated light scattering. On the basis of this data we expect the phenol–triazole ligands to have the appropriate Cu-binding affinity to interact with Cu in the presence of the Aβ peptide.

**Table 1:** Stability constant measurements (log *K*'s) of each ligand with Cu^2+^

Reaction	log *K*		
	**POH**	**PMorph**	**PTMorph**
Cu^2+^ + L^–^ = [CuL]^+^	8.97(2)	8.90(3)	8.77(7)
Cu^2+^ + 2L^–^ = [CuL_2_]	15.46(3)	15.65(2)	15.16(9)
[CuL_2_(H_2_O)] = [CuL_2_(OH)]^–^ + H^+^	–14.36(3)	–13.98(9)	–12.96(2)
pCu^*a*^	6.9	6.8	6.6

^*a*^pCu was calculated using pCu = (–log[Cu^2+^]_free_), where [Cu^2+^]_free_ is determined from the HySS model.^45^

### Anti-oxidant assays

**Fig. 3:**
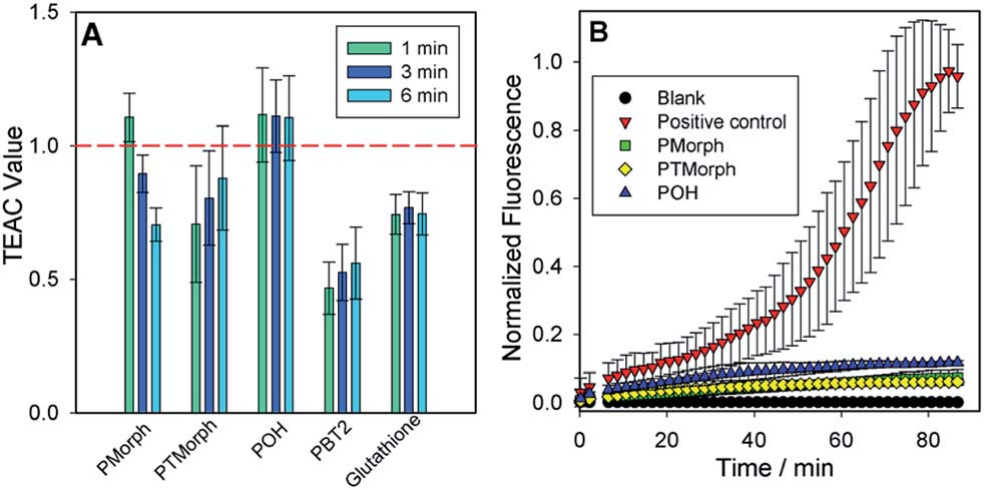
(A) Trolox-Equivalent Anti-oxidant Capacity (TEAC) values at 1, 3, and 6 minutes for Trolox, PBT2, glutathione, and each phenol–triazole ligand. Each experiment was completed in triplicate and error bars represent ±SD for the average TEAC values. Note: PBT2 shows weak anti-oxidant capacity in TEAC assay most likely due to electron-withdrawing chloride substituents in the *ortho*/*para* positions. (B) A fluorogenic assay monitoring the production of 7-hydroxyCCA over time. In the absence of any exogenous ligands, free Cu exhibits Fenton-like chemistry to produce hydroxyl radicals, which react with CCA to form a fluorescent product. Introduction of **POH**, **PMorph**, or **PTMorph** to a solution containing CCA, Cu, and ascorbate, a distinct inhibition in fluorescent product is observed. Conditions: 40 μM CuSO_4_, 100 μM CCA, 80 μM ligands, 400 μM l-ascorbic acid, *λ*_ex_: 395 nm/*λ*_em_: 450 nm.

To further evaluate the multifunctional nature of the phenol–triazoles, we investigated their antioxidant capability using several different assays.^46–48^ In the first assay, the antioxidant activity of the phenolic functions were evaluated using a Trolox-Equivalent Antioxidant Capacity (TEAC) assay. The TEAC assay has been used to quantify the antioxidant activity of biological fluids, extracts, and pure compounds by measuring the disappearance of the ABTS^+^˙ radical cation *via* UV-vis spectroscopy.^49^ Trolox, a water-soluble vitamin-E analogue, is used as a standard against which each ligand is compared. In addition, each phenol–triazole ligand was compared with glutathione and PBT2, an 8-hydroxyquinoline derivative, that has shown promise as an Alzheimer's disease therapeutic.^50,51^ Each ligand exhibited TEAC values comparable to Trolox and slightly enhanced in comparison to PBT2 (Fig. 3).

In two other complimentary antioxidant assays, we measured the ability of the phenol–triazole ligands to bind Cu and limit hydroxyl radical formation from aqueous Cu in the presence of dioxygen. Firstly, using a fluorescent coumarin carboxylic acid (CCA) assay, in which the highly reactive hydroxyl radical specifically hydroxylates CCA at the 7-position to form a fluorescent product,^52,53^ aerobic aqueous Cu solutions in the presence of a physiologically-relevant concentration of ascorbate^54^ exhibit significant fluorescence over a short period (Fig. 3). The addition of 2 eq. **POH**, **PMorph**, or **PTMorph** to the Cu/ascorbate/CCA solutions significantly reduces the observed fluorescence (Fig. 3), consistent with either the high affinity of these ligands for Cu (*vide supra*), or direct reaction of the ligands with the generated ˙OH. A complimentary ascorbate (Asc) reduction assay^55,56^ was employed to discern if Cu chelation was responsible for the observed response in the CCA experiments. In the absence of ligands, Asc consumption in the presence of Cu is rapid (Fig. S10†), however, upon addition of 3 eq. of the high affinity chelator DTPA, Asc consumption is arrested. We observed only a small decrease in Asc consumption upon addition of 10 eq. of phenol–triazole ligands to a Cu/Asc system (Fig. S10†), signifying that the compounds act primarily as antioxidants, and not *via* Cu redox silencing, in the CCA assay. Ascorbate consumption was also evaluated in the presence of Aβ_1–16_, which contains the metal binding region for Cu (Fig. S11†). When comparing Cu–Aβ_1–16_ *vs.* Cu–Aβ_1–16_ + 10 eq. of phenol–triazole ligand, no change in the rate of ascorbate consumption was observed. This suggests that the ligands do not limit Cu^2+/+^ redox cycling and therefore, act primarily as antioxidants, reacting with Cu-generated ROS.

### Ligand–Aβ peptide interactions

The direct interaction of the phenol–triazole ligands with monomeric Aβ_1–40_ was investigated using 2-D ^1^H–^15^N SOFAST-HMQC NMR experiments.^29,57^ The less aggregation prone Aβ_1–40_ peptide length was used here to limit aggregation during data collection and ensure all shifts are solely the result of peptide–ligand interactions and not peptide aggregation. Incubation with one of **POH**, **PMorph**, or **PTMorph** resulted in small but detectable chemical shift changes for specific peptide residues (see ESI for experimental details†).

**Fig. 4:**
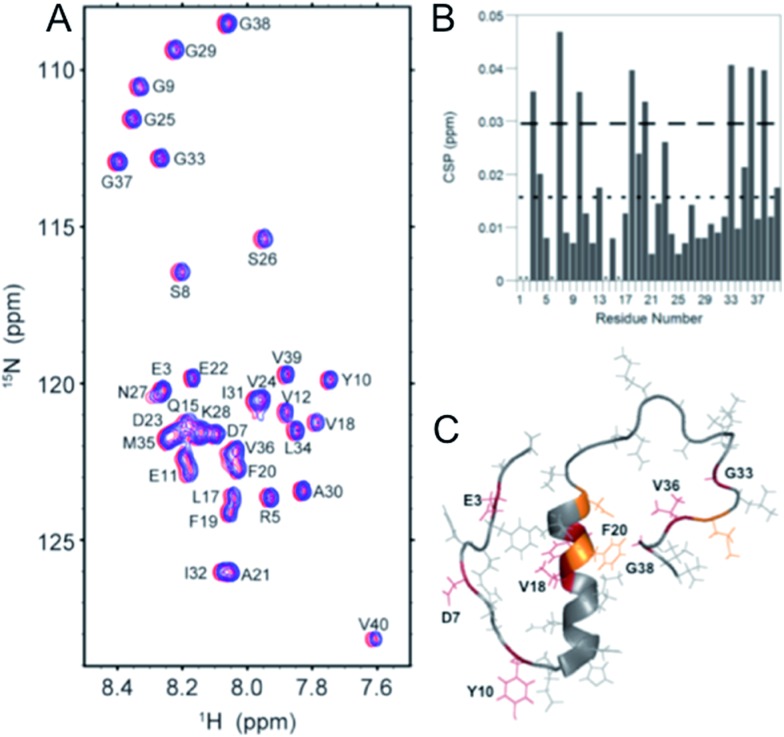
2-D ^1^H–^15^N SOFAST NMR experiments using ^15^N-labeled Aβ_1–40_ and 0–10 eq. **POH**. (A) 2-D ^1^H–^15^N SOFAST NMR spectra showing the assignment of specific amino acid residues in the Aβ_1–40_ peptide. (B) Summary of the specific amino acid residues that have shifted at 10 eq. **POH**. The dotted line represents the average CSP while the dashed line is the average + one standard deviation, which was used to identify statistically relevant Chemical Shift Perturbations (CSP). (C) Aβ_1–40_ solution NMR structure (PDB: ; 2LFM) highlighting specific amino acid residues that have significant CSP shifts (Red, >0.03 ppm shift) and moderate CSP shifts (orange, between 0.02 and 0.03 ppm).

Interestingly, **POH** was determined to exhibit significant chemical shift changes distributed across the entire peptide length, including Aβ residues E3, D7, Y10, V18, F20, G33, V36, and G38 (Fig. 4). In the case of **PMorph**, significant chemical shift changes were observed for Aβ residues D7, F19, D23, and N27 (Fig. S12†). The hydrophobic region of the Aβ peptide, encompassing residues 17–21, and in particular F19, is considered to play a critical role in the initial stages of peptide aggregation, and thus the interaction of **PMorph** with F19 may play a significant role in mediating the Aβ aggregation process (*vide infra*).^58–63^ **PTMorph** demonstrates similar interactions to those observed with **PMorph**, including significant chemical shift changes of Aβ residues D7, V18, F19, N27, and G33 (Fig. S13†). The similar interaction of **PMorph** and **PTMorph** with Aβ is not surprising as the only structural difference is the O for S heteroatom substitution.

Although limited to three ligands, the 2-D NMR results suggest that the triazole R-group plays a significant role in dictating Aβ peptide interactions (Fig. S14†), with the propanol group of **POH** affording non-specific interactions, while the morpholine and thiomorpholine heterocycles confer a higher degree of selectivity for Aβ peptide residues. The direct interaction of the phenol–triazoles with the Aβ peptide, as supported by the 2-D NMR studies, suggest that these ligands may modulate the Aβ aggregation profile in solution, even in the absence of Cu (*vide infra*).

### Aβ peptide aggregation experiments

Modulation of the Aβ peptide aggregation pathway offers a significant opportunity for drug development. Shifting the Aβ aggregation pathway away from toxic intermediates, such as soluble oligomeric species,^64–68^ may lead to a decrease in neuronal cell death.

**Fig. 5:**
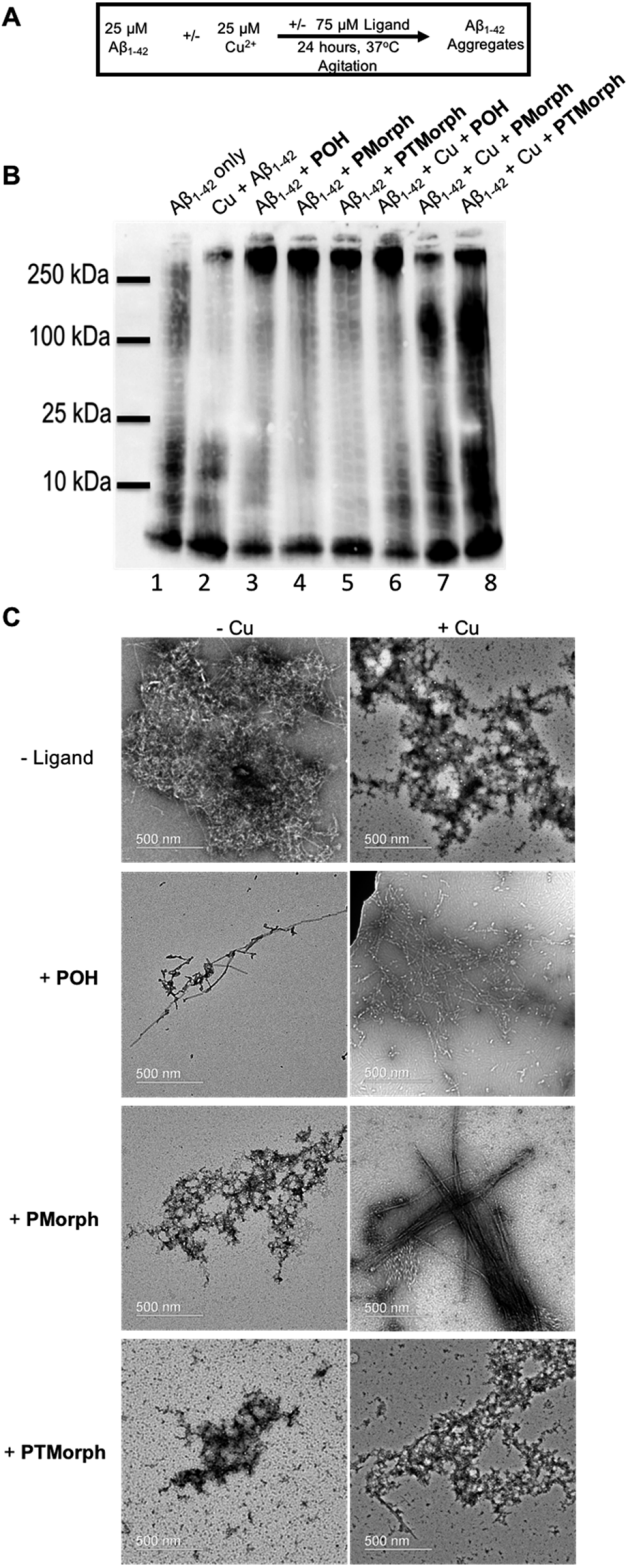
Influence of **POH**, **PMorph**, **PTMorph** on the aggregation profile of Aβ_1–42_ ± Cu^2+^ (A). Experimental aggregation scheme (B). Native gel electrophoresis of 25 μM Aβ_1–42_ ± 1 eq. CuCl_2_ and ±3 eq. ligand using anti-Aβ antibody 6E10. Lane 1: 25 μM Aβ_1–42_ only; lane 2: 25 μM Aβ_1–42_ + 1 eq. CuCl_2_; lane 3: 25 μM Aβ_1–42_ + 3 eq. **POH**; lane 4: 25 μM Aβ_1–42_ + 3 eq. **PMorph**; lane 5: 25 μM Aβ_1–42_ + 3 eq. **PTMorph**; lane 6: 25 μM Aβ_1–42_ + 1 eq. CuCl_2_ + 3 eq. **POH**; lane 7: 25 μM Aβ_1–42_ + 1 eq. CuCl_2_ + 3 eq. **PMorph**; lane 8: 25 μM Aβ_1–42_ + 1 eq. CuCl_2_ + 3 eq. **PTMorph**. (C) TEM images of Aβ_1–42_ post-24 hour incubation under conditions detailed in (B).

To further explore the multifunctional nature of the phenol–triazole ligands we investigated the effects of these ligands on Aβ peptide aggregation in the presence and absence of Cu ions (Fig. 5A). Using native gel electrophoresis/western blotting to visualize the size distribution of Aβ species, in conjunction with transmission electron microscopy (TEM) to examine Aβ aggregate morphology, we assessed the ability of the phenol–triazole ligands to modulate Aβ peptide aggregation.

Incubation of the phenol–triazole ligands with the Aβ peptide results in significant changes to the size distribution of peptide aggregates (Fig. 5B), highlighting the importance of the interactions observed in the 2-D NMR experiments. Incubation of Aβ_1–42_ alone over 24 h (lane 1) affords both high and low molecular weight species, and significant fibril formation as observed by TEM (Fig. 5C), in line with previous reports.^42,69,70^ Addition of the phenol–triazole ligands significantly alters the aggregation profile towards high molecular weight species (Fig. 5B, lanes, 3, 4 and 5), and in addition the aggregate morphology is now amorphous as ascertained by TEM (Fig. 5C). These results show that the presence of the phenol–triazole ligands limits the formation of oligomeric species (10–100 kDa range), which have been implicated to play a major role in Aβ-associated toxicity.^71,72^

We next investigated the effect of the phenol–triazole ligands on Aβ peptide aggregation in the presence of Cu ions. The metal competition assays (Fig. S7–S9†) suggest that the phenol–triazole ligands only partially demetallate Cu–Aβ and thus we investigated the effect of the ligands on aggregation of metalated peptide species. Incubation of Aβ_1–42_ with 1 eq. of Cu affords primarily oligomeric species in comparison to Aβ_1–42_ alone at 24 h (Fig. 5B, lane 1 *vs.* 2).^15^ In addition, aggregate morphology has changed from fibrillar to amorphous (Fig. 5C). Incubation of Cu–Aβ peptide solutions with the phenol–triazole ligands leads to significant changes in the aggregation profile (Fig. 5B, lane 2, *vs.* lanes 6, 7, and 8). For **POH**, a significant shift towards high molecular weight species is observed, with a similar soluble aggregate profile to that observed in the peptide only experiment (Fig. 5B, lane 3 *vs.* lane 6). Interestingly, TEM analysis shows primarily fibrillar aggregates for Cu–Aβ in the presence of **POH** (Fig. 5C). While this data is consistent with Cu sequestration by the ligand and aggregation of the peptide,^73^ it is likely that ligand–peptide interactions are the dominant factor that controls the aggregation process of both Aβ_1–42_ and Cu–Aβ_1–42_.

For **PMorph** and **PTMorph**, similar Aβ-aggregation profiles are observed in the presence of Cu (Fig. 5B, lanes 7 and 8), which differ significantly from the Cu–Aβ experiment (Fig. 5B, lane 2), and also from the **POH** experiment (Fig. 5, lane 6). Interestingly, **PMorph** and **PTMorph** shift Aβ aggregation towards distinctly different high molecular weight species in comparison to **POH** (*ca.* 100 kDa *vs.* 250 kDa). TEM analysis of the Cu–Aβ aggregation experiment in the presence of **PMorph** shows the formation of fibrils (Fig. 5C), while for **PTMorph** both fibrils and amorphous aggregates are observed (Fig. 5C). The three phenol–triazole ligands exhibit similar Cu affinity constants and thus the different aggregation profiles observed for the ligands in the presence of Cu–Aβ may result from distinct interactions of the triazole R-groups with the Aβ peptide, leading to ternary complex formation,^19,74,75^ and/or interactions with specific aggregates, such as oligomeric Aβ.

### Attenuation of Aβ-induced neurotoxicity in human neuronal culture

**Fig. 6:**
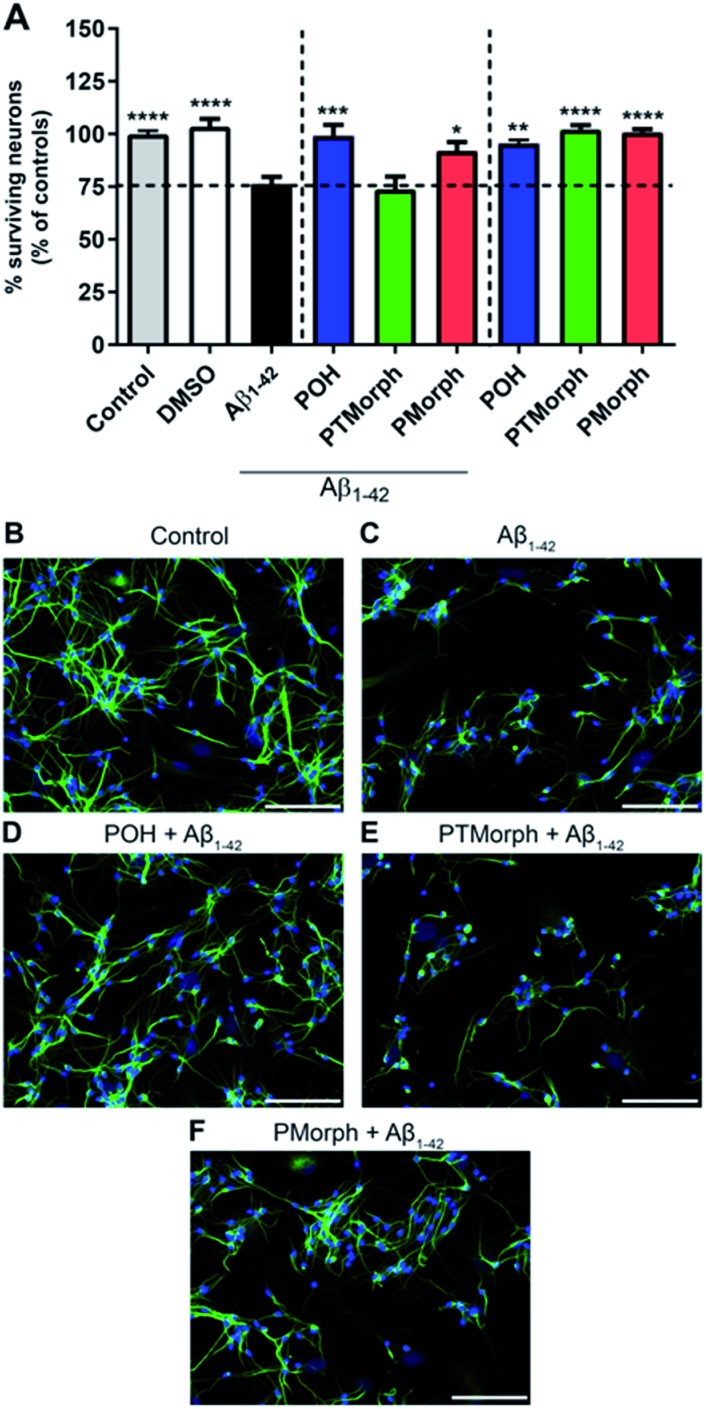
(A, C) Treatment of human fetal neurons (HFN) with 25 μM Aβ_1–42_ induced cell death after 24 hours compared to the control condition (B). One hour pre-incubation with 75 μM **POH** prevented Aβ_1–42_-induced neurotoxicity (*p* < 0.001, (D)). **PMorph** was also shown to be neuroprotective (*p* < 0.05, (E)), whereas **PTMorph** had no effect (F). Treatment of HFN with ligands in the absence of Aβ_1–42_ did not induce cell death (A). Pictures are shown at 20× original magnification, scale bar = 100 μm. Significance is shown compared to HFN with Aβ_1–42_. *p < 0.05, **p < 0.01, ***p < 0.001, ****p < 0.0001.

To investigate whether the phenol–triazole ligands protect against Aβ_1–42_-mediated neurotoxicity, human fetal neurons (HFN) were treated with **POH**, **PTMorph** and **PMorph** for 1 hour prior to Aβ_1–42_ application. Treatment with monomeric Aβ_1–42_ induced significant cell death (Fig. 6, 24% fewer MAP-2 positive neurons compared to the control condition, *p* < 0.0001). Upon treatment with 75 μM **POH**, complete prevention of Aβ_1–42_-induced neurotoxicity was achieved (Fig. 6, *p* < 0.001). **PMorph** was also able to protect against Aβ_1–42_-induced neurotoxicity (*p* < 0.05), while **PTMorph** demonstrated no protective effect in comparison to Aβ_1–42_. When 75 μM of ligand was incubated with HFN for 24 hours in the absence of any Aβ_1–42_, similar cell viability was observed in comparison to the control, indicating that these ligands are not toxic at the experimental concentration used.

We next assessed the neurotoxicity of Aβ_1–42_ premixed with Cu (1 : 1) at 5, 10 and 25 μM (Fig. S15†). While Cu alone was not toxic at these concentrations, Cu–Aβ_1–42_ exhibited significant toxicity at all concentrations studied. Pre-incubation of the HFN with the phenol–triazole ligands before addition of Cu–Aβ_1–42_ did not have a neuroprotective effect at the 24 h timepoint (Fig. S15†). Overall, these results are consistent with the previously reported toxicity of Cu–Aβ_1–42_ species,^15,76,77^ and show that under the HFN assay conditions the phenol–triazole ligands cannot prevent Cu–Aβ_1–42_ toxicity. While not effective in limiting the toxicity of Cu–Aβ_1–42_ in neurons, the phenol–triazole ligands are effective under metal-free conditions. Overall, **POH** demonstrates the best neuroprotective properties when in the presence of Aβ_1–42_, conferring complete protection, followed by **PMorph**, with **PTMorph** being ineffective in this assay.

## Summary

In this report, we describe the development of a series of phenol–triazole ligands that target multiple factors associated with AD etiology. The modular synthetic strategy afforded ligands that exhibit favourable physicochemical properties *via* both experiments (p*K*_a_ values) and calculations (drug-likeness), while also displaying antioxidant activity. The three phenol–triazole ligands were shown to interact with the Aβ peptide *via* 2-D NMR studies, with the **PMorph** and **PTMorph** derivatives displaying similar interactions with specific peptide residues, including F19. Interestingly, **POH** displays a larger number of interactions distributed across the length of the Aβ peptide. The three ligands alter the Aβ peptide aggregation profile in a similar manner *via* limiting oligomer formation in favour of amorphous high molecular weight species. The Cu-binding affinity of the phenol–triazoles was determined to be of appropriate strength to compete with the Aβ peptide, and as expected the ligands altered the Cu–Aβ aggregation profile. In the presence of Cu, only **POH** significantly reduced Aβ oligomer formation promoting the formation of large molecular weight aggregates. These results highlight that the triazole R-group offers a significant opportunity to tune the biological properties of the ligand scaffold. Encouragingly, **POH**, and to a lesser extent **PMorph**, exhibited a protective effect against Aβ_1–42_-induced neurotoxicity in human neuronal culture, while these ligands were not able to rescue the neurotoxicity associated with Cu–Aβ_1–42_ under our conditions. Altogether, these promising results strongly suggest that the multifunctional phenol–triazole ligand scaffold, and in particular **POH**, warrants further investigation in an animal model to interrogate mechanisms of action and efficacy.
