# Supplementary material for: Multi-target-directed phenol–triazole ligands as therapeutic agents for Alzheimer's disease
Source: Chem Sci. 2017 Jun 5;8(8):5636–43. doi: 10.1039/c7sc01269a (PMC5621006; doi:10.1039/c7sc01269a)
Supplement: Supplementary file 1 [file SC-008-C7SC01269A-s001.pdf]

Electronic Supplementary Material (ESI) for Chemical Science.
This journal is © The Royal Society of Chemistry 2017

**Supplementary Material**

# Multitarget-Directed Phenol-Triazole Ligands as Therapeutic Agents for Alzheimer's Disease

*Michael R. Jones[a,b]; Emilie Mathieu[a]; Christine Dyrager[a]; Simon Faissner[b,c]; Zavier Vaillancourt[a]; Kyle Korshavn[d]; Mi Hee Lim[e]; Ayyalusamy Ramamoorthy[d,f]; V. Wee Yong[b]; Shigeki Tsutsui[b]; Peter Stys[b]; Tim Storr[a]**

[a]*Department of Chemistry, Simon Fraser University, V5A-1S6, Burnaby, BC, Canada*

[b]*Department of Clinical Neurosciences, Hotchkiss Brain Institute, Cumming School of Medicine, University of Calgary, Calgary, Canada*

[c]*Department of Neurology, St. Josef-Hospital, Ruhr-University, Bochum, Germany*

[d]*Department of Chemistry, University of Michigan, Ann Arbor, USA*

[e]*Department of Chemistry, Ulsan National Institute of Science and Technology (UNIST), Ulsan, Korea*

[f]*Department of Biophysics, University of Michigan, Ann Arbor, USA*

*Email: tim_storr@sfu.ca

**Experimental Section**

**Materials and Methods.** All reagents were purchased as reagent grade from commercial suppliers and used without further purification unless otherwise indicated. 4-(2-azidoethyl)morpholine[1], 4-(2-azidoethyl)thiomorpholine[2], 3-azidopropan-1-ol,[3] and 2-((trimethylsilyl)ethynyl)phenol[4] were synthesized according to previously reported procedures. ***Safety Precautions in Handling of Azides:*** Some azides are hazardous, and to avoid injury, follow safe laboratory practices, wear appropriate protective equipment and use appropriate shielding equipment. Azides can decompose violently upon heating, shock and/or friction. Only small quantities should be prepared at a single time. $A\beta_{1-42}$ peptide was purchased from 21st Century Biochemicals (Marlborough, MA, USA). $^{15}$N-labeled $A\beta_{1-40}$ peptide was purchased from rPeptide. The gels were purchased from BioRad and membranes from PALL – Life Sciences. $^{1}$H, $^{13}$C, and 2-D $^{1}$H-$^{15}$N SOFAST HMQC NMR spectra were recorded on a 600 MHz Bruker Avance NMR spectrometer. Mass spectra (positive ion) were obtained on an Agilent 6210 time-of-flight electrospray ionization mass spectrometer. High-resolution electrospray ionization mass spectrometry (HRMS-ESI+) was performed at the Mass Spectrometry and Proteomics Facility at the University of Notre Dame. Electronic spectra were obtained on a Cary 5000 spectrophotometer. FT-IR spectra were obtained using a Thermo Nicolet Nexus 670 FT-IR spectrophotometer equipped with a Pike MIRacle attenuated total reflection (ATR) sampling accessory.

**Synthesis.**

*2-(1-(3-hydroxypropyl)-1H-1,2,3-triazol-4-yl)phenol* (**POH**). To a solution of 2-((trimethylsilyl)ethynyl)phenol[4] (0.195 g, 1.02 mmol) and 3-azidopropan-1-ol[3] (0.113 g,

1.11 mmol) in methanol (6 mL) was added a solution of $CuSO_4 \bullet 5H_2O$ (0.130 g, 0.52 mmol), *L*-ascorbic acid (0.072 g, 0.41 mmol), and $K_2CO_3$ (0.258 g, 1.87 mmol) in water (6 mL), while stirring vigorously. Pyridine (0.4 mL, 4.97 mmol) was added slowly to the resulting mixture and the solution was stirred at room temperature overnight. Chelex® was added and the solution was stirred for an additional 2 hours. The solution was filtered, concentrated *in vacuo* and redissolved in $H_2O$ (1 mL). The aqueous layer was extracted with dichloromethane (3 x 5 mL), the organic layers were combined, dried over $Na_2SO_4$ and concentrated *in vacuo* and purified by column chromatography (100% EtOAc) affording **POH** as an off-white powder (0.054 g, 24% yield). $^1$H NMR ($CDCl_3$, 400 MHz): δ 10.83 (s, 1H), 7.88 (s, 1H), 7.42 (dd, *J* = 7.8, 1.5 Hz, 1H), 7.22-7.26 (m, 1H), 7.06 (dd, *J* = 8.3, 1.0 Hz, 1H), 6.90 (td, *J* = 7.7, 1.2 Hz, 1H), 4.60 (t, *J* = 6.8 Hz, 2H), 3.68 (t, *J* = 5.8 Hz, 2H), 2.19 (q, *J* = 6.3 Hz, 2H). $^{13}$C NMR ($CDCl_3$, 151 MHz): δ 156.1, 147.7, 129.9, 126.0, 119.7, 119.6, 117.9, 114.1, 58.9, 47.5, 32.6. HRMS-ESI+ (*m/z*) : $[M + H]^+$ calculated for ($C_{11}H_{14}N_3O_2$), 220.1081; Found, 220.1103.

*2-(1-(2-morpholinoethyl)-1H-1,2,3-triazol-4-yl)phenol* **(PMorph).** To a solution of 2-((trimethylsilyl)ethynyl)phenol[4] (0.195 g, 1.02 mmol) and 4-(2-azidoethyl)morpholine[1] (0.176 g, 1.13 mmol) in methanol (6 mL) was added a solution of $CuSO_4 \bullet 5H_2O$ (0.130 g, 0.52 mmol), *L*-ascorbic acid (0.072 g, 0.4 mmol), and $K_2CO_3$ (0.258 g, 1.87 mmol) in water (6 mL), while stirring vigorously. Pyridine (0.4 mL, 4.97 mmol) was added slowly to the resulting mixture and the solution was stirred at room temperature for 16 hours using similar conditions to the synthesis of **POH**. The crude residue was purified by column chromatography (dichloromethane/MeOH 95:5) affording **PMorph**

as a light yellow powder (0.106 g, 38% yield). $^{1}H$ NMR ($CDCl_3$, 400 MHz): δ 10.84 (s, 1H), 7.99 (s, 1H), 7.42 (dd, $J$ = 7.7, 1.6 Hz, 1H), 7.22-7.27 (m, 1H), 7.05 (dd, $J$ = 8.3, 1.1 Hz, 1H), 6.91 (td, $J$ = 7.5, 1.2 Hz, 1H), 4.55 (t, $J$ = 6.4 Hz, 2H), 3.71 (t, $J$ = 4.7 Hz, 4H), 2.89 (t, $J$ = 6.3 Hz, 2H), 2.53 (t, $J$ = 4.1 Hz, 4H). $^{13}C$ NMR ($CDCl_3$, 151 MHz): δ 156.04, 147.8, 129.86, 125.92, 119.61, 119.57, 117.86, 114.16, 66.99, 57.95, 53.67, 47.92. HRMS-ESI+ (*m/z*): $[M + H]^+$ calculated for ($C_{14}H_{19}N_4O_2$), 275.1503 Found, 275.1536.

*2-(1-(2-thiomorpholinoethyl)-1H-1,2,3-triazol-4-yl)phenol* **(PTMorph).** To a solution of 2-((trimethylsilyl)ethynyl)phenol[4] (0.195 g, 1.02 mmol) and 4-(2-azidoethyl)thiomorpholine[2] (0.200 g, 1.16 mmol) in methanol (6 mL) was added a solution of $CuSO_4 \bullet 5H_2O$ (0.130 g, 0.52 mmol), *L*-ascorbic acid (0.072 g, 0.41 mmol)), and $K_2CO_3$ (0.258 g, 1.87 mmol) in water (6 mL), while stirring vigorously. Pyridine (0.4 mL, 4.97 mmol) was added slowly to the resulting mixture and was stirred at room temperature using similar conditions to the synthesis of **POH**. The crude residue was purified by column chromatography (dichloromethane/MeOH 95:5) affording **PTMorph** as a brownish-yellow powder (0.095 g, 42% yield). $^{1}H$ NMR ($CDCl_3$, 400 MHz): δ 10.83 (s, 1H), 7.98 (s, 1H), 7.42 (dd, $J$ = 7.7, 1.6 Hz, 1H), 7.23-7.28 (m, 1H), 7.06 (dd, $J$ = 8.3, 1.0 Hz, 1H), 6.92 (td, $J$ = 7.6, 1.2 Hz, 1H), 4.51 (t, $J$ = 6.2, 2H), 2.90 (t, $J$ =6.3, 2H), 2.80 (t, $J$ = 4.3, 4H), 2.67 (t, $J$ = 4.4, 4H). $^{13}C$ NMR ($CDCl_3$, 151 MHz): δ 155.95, 147.61, 129.79, 125.93, 119.63, 119.61, 117.87, 114.15, 58.20, 55.10, 48.18, 28.09. HRMS-ESI+ (m/z) : $[M + H]^+$ calculated for ($C_{14}H_{19}N_4OS$), 291.1274; Found, 291.1314.

**Predictability of Drug-Like/BBB Permeability Properties**. Using the website molinspiration.com, several physicochemical properties were determined in order to predict the drug-like properties and blood-brain barrier (BBB) permeability of each ligand. cLogP, $M_w$, total potential surface area (TPSA), H-bond acceptors, and H-bond donors were calculated to determine the drug-like properties of each ligand.[5] Clark's equation[6] (**Eq. S1**, logBB = -0.0148 Å x TPSA + 0.152 Å x MiLogP + 0.139) was used to determine logBB, which is a reasonable indication of permeability through the blood-brain barrier.

**Acidity Constant Determination via UV-vis and $^1$H NMR Spectroscopies.** In order to determine the speciation of **POH**, **PMorph**, and **PTMorph** at physiological pH, acidity constants were measured by obtaining variable pH UV-vis spectra. Solutions of all three ligands (50 μM) were prepared in 0.1 M NaCl(aq.) at pH 3 using concentrated HCl. Before obtaining UV-vis spectra, a pH electrode was calibrated using a 2-point method (pH 4.01 and pH 10.01 standard buffers). NaOH was used to increase the pH of the ligand solutions to obtain at least 30 spectra ranging from pH 3 – 12 from 600 – 190 nm. Spectral data was tabulated and analyzed using the HypSpec program (Protonic Software, UK).[7] A model was created for each ligand and simulated to fit the experimental data using selected wavelengths where significant spectral changes were observed. To complete the speciation diagrams for these 3 ligands, variable pH $^1$H NMR spectroscopy was also employed in order to probe regions of the molecule that do not undergo p$K_a$ -dependent UV-vis changes. Solutions of 80 – 100 mg ligand in 8 mL of $D_2O$ were prepared. NaOD and DCl were used to vary the pD of the solutions. Once the pH of the

solution had stabilized, 500 μL of solution was placed in an NMR tube. Seven aliquots were prepared and $^1$H NMR experiments were run on a Bruker AVANCE 600 MHz NMR. Depending on the R-group, the $pK_a$ values were determined by measuring the chemical shifts of the protons in the vicinity of the protonation site over a specific pH range. Data obtained was tabulated and analyzed using HypNMR (Protonic Software, UK).[7] The equation ($pK_a$ ($H_2O$) = ($pK_a$ ($D_2O$) – 0.45)/1.015) was used to convert pD values to pH values. Speciation diagrams for **POH**, **PMorph**, **PTMorph** were simulated using the HySS2009 program (Protonic Software, UK).[8]

**Metal Stability Constant Measurements**. Metal stability constant measurements were performed by running spectra of 75 μM ligand + 37.5 μM $CuCl_2$. The solution was subjected to variable pH measurements using HCl and NaOH in the range pH 3-11. Approximately 30 UV-vis spectra were obtained over the pH range. The UV-vis spectra were tabulated and HypSpec was used to analyze the data.[7] A model of the potential species was developed and simulated to obtain the best fit to the experimental data.

**Cu Competition Assay.** UV-Visible spectra were obtained from 260-350 nm in order to determine if the phenol-triazole series of ligands could compete with either $A\beta_{1-42/1-16}$ for binding Cu. Stock solutions were prepared using Chelex-treated 50 mM HEPES buffer pH 7.4. Prior to phenol-triazole addition, Cu-Aβ solutions were allowed to incubate for 20 mins. at 37 °C to ensure complete Cu binding by the peptide. Final concentrations were as follows: $CuSO_4$ – 37.5 μM; Phenol-triazole ligands – 75 μM; $A\beta_{1-42/1-16}$ – 45 μM.

**Job Plot Analysis**. To determine the ligand:Cu stoichiometry for the phenol-triazole series of ligands, a stock solution (0.7 mM) of **PMorph** and $CuCl_2$ (1 mM) were prepared in PBS pH 7.4 buffer and spectra were measured using a Cary UV-Vis-NIR instrument. Each phenol-triazole ligand has the same metal binding unit, therefore each ligand is assumed to bind to Cu in a similar manner. Solutions containing different ratios of ligand and Cu ions were recorded from 0 to 100 mol % Cu (total concentration = 100 μM). Appropriate amounts of the stock solutions were dissolved into 1 mL of PBS buffer and allowed to equilibrate for 5 minutes before recording the spectra. The absorption at 320 nm was plotted as a function of the mole fraction Cu (Cu).

**Coumarin Carboxyic Acid (CCA) Antioxidant Assay:** Cu ions in an aqueous environment can react with $O_2$(g) to produce reactive oxygen species such as the hydroxyl radical ($OH^{\bullet}$) and, in the presence of a reducing agent (i.e: ascorbic acid), this process can become catalytic.[9] The non-fluorescent coumarin-3-carboxylic acid (CCA) reacts with hydroxyl radicals to produce the fluorescent 7-hydroxycoumarin-3-carboxylic acid.[9] The production of the fluorophore can be monitored to evaluate the efficiency of ligands to bind Cu, and therefore limit hydroxyl radical formation. This assay was adapted to be performed in a 96-well plate.[10] Stock solutions (10X concentrations) were prepared in $H_2O$ and added to each well in this order with the following final concentrations: $CuSO_4$ (40 μM), CCA (100 μM), ligands (80 μM), and *L*-ascorbic acid (400 μM). Final volume in each well was 200 μL, which was constituted up using pH 7.4 PBS buffer. Fluorophore formation was monitored by $\lambda_{ex}$: 395 nm and $\lambda_{em}$: 450 nm. Each

experiment was performed in quadruplicate and allowed to react for 75 minutes. Blanks consisted of CCA, ligand, *L*-ascorbic acid, and pH 7.4 PBS buffer.

**Trolox Equivalent Antioxidant Capacity (TEAC) Assay:**

The method described by Rice-Evans *et al.* was adapted to a plate reader in order to perform several experiments simultaneously.[11] First, ABTS (0.0082 g, 2.7 eq.) and $K_2S_2O_8$ (0.0016 g, 1 eq.) were dissolved in deionized water (2 mL) to form $ABTS^{+\bullet}$. The components were allowed to react for 16 hours in the dark at room temperature. An $ABTS^{+\bullet}$ concentration exhibiting an absorbance value around 0.7 was achieved by aliquoting 3-42 μL $ABTS^{+\bullet}$ solution into a transparent 96-well plate dissolved in MeOH up to a final volume of 300 μL. Each volume was measured in triplicate at 470 nm to determine which volume had an absorbance at 0.7 using a Synergy 4 Fluorometer plate reader from BioTek, and plotted as a function of ABTS concentration. The absorbance increased linearly in this range and a volume of 30 μL was chosen for the next step as this corresponded to an absorbance measurement of 0.7 at 470 nm. The TEAC values of the ligands, Trolox, PBT2 and glutathione were determined. Stock solutions of the compounds in MeOH (1.5 mM) were prepared to final concentrations of 25, 50, 75, 100, and 125 μM and added into a transparent 96-well plate, then diluted with MeOH up to a volume of 270 μL. Just before the measurement, 30 μL of the $ABTS^{+\bullet}$ solution was added into each well with a multichannel pipette. The plate was shaken for 30 seconds, and the absorbance at 470 nm was recorded every minute for 6 minutes using a Synergy 4 Fluorometer plate reader from BioTek. Each measurement was performed in triplicate. The value of the absorbance was plotted as a function of the concentration of compound

at a set time and the slopes obtained were then compared to that of Trolox, which was normalized to 1, to give the TEAC value at 1, 3 and 6 minutes.

**Cu-induced Ascorbate Consumption Assay:** A 1 mM stock solution of ascorbate was prepared in 50 mM HEPES buffer pH 7.4 along with a 3 mM stock solution of $CuSO_4$ and 900 μM stock solutions of each phenol-triazole ligand. Final concentrations in the assay were as follows: 100 μM Ascorbate, 6 μM $CuSO_4$, 60 μM phenol-triazole ligand, 7.2 μM $A\beta_{1\text{-}16}$ (1.2 eq. compared to $CuSO_4$), final volume in cuvette was 1 mLl. A 3 mL quartz cuvette was loaded with ascorbate and HEPES buffer and the reaction was initiated by addition of the Cu stock solution. Once the absorbance at 265 nm had reached ca. 0.8, the ligand stock solution was added in a single volume and monitored for 30 mins. For the $A\beta_{1\text{-}16}$ experiments, a quartz cuvette was loaded with ascorbate, HEPES buffer, and 1.2 eq. $A\beta_{1\text{-}16}$ (compared to Cu). Cu was added to the solution and the absorbance at 265 nm was monitored until it reached 0.8, where a single volume of the ligand stock solution was added. The reaction was monitored for 30 mins.

**2-D SOFAST HMQC NMR Spectroscopy**. 2-D band-Selective Optimized Flip Angle Short Transient (SOFAST) Heteronuclear Multiple Quantum Correlation (HMQC) NMR experiments provide rapid residue-specific insight into the protein environment. NMR samples were prepared from $^{15}N$-labeled $A\beta_{1\text{-}40}$ (rPeptide) by first dissolving the peptide in 1% $NH_4OH_{(aq)}$ and lyophilizing to remove preformed aggregates. The peptide was re-dissolved in 3 μL of DMSO-$d_6$ and diluted into buffer for a final peptide concentration of 80 μM (pH 7.4, 20 mM $PO_4$, 50 mM NaCl, 7% $D_2O$ v/v, 1% DMSO v/v). Spectra were

taken at each titration point using a 600 MHz Bruker Avance NMR spectrometer equipped with a triple-resonance z-gradient cryogenic probe at 8 °C with 128 $t_1$ experiments, 128 scans, and a 100 ms recycle delay. The 2D $^{15}N$-$^{1}H$ SOFAST-HMQC data were processed using TOPSPIN 2.1 (Bruker) and resonance assignment was performed with Sparky 3.1134. Resonances were assigned based on previous assignments under similar conditions.[12-14] Chemical shift pertubations were calculated using equation S2:

**Eq. S2:**

$$\Delta\delta_{NH} = \sqrt{(\Delta\delta_H{}^2) + \left(\frac{\Delta\delta_N}{5}\right)^2}$$

where $\Delta\delta_H$ is defined as the change in $^{1}H$ chemical shift and $\Delta\delta_N$ is defined as the change in the $^{15}N$ chemical shift upon addition of ligand to the $^{15}N$-labeled $A\beta_{1-40}$ relative to the chemical shifts of peptide in the absence of ligand.

**Aβ Peptide Aggregation Experiments.** The ability of **POH**, **PMorph**, and **PTMorph** to influence the aggregation of $A\beta_{1-42}$ was further evaluated by molecular weight separation on a 10 – 20% gradient tris-tricine gel and visualized using western blotting techniques. Each sample was incubated for 24 hours under constant agitation at 37 °C in a 96-well plate, covered with a lid and sealed with parafilm. Final concentrations, diluted in PBS pH 7.4, were as follows: 25 μM $A\beta_{1-42}$, 25 μM $CuCl_2$, 75 μM (3 eq.) ligand. Samples were loaded onto a 10-20 % gradient tris-tricine gel (Bio-Rad #456-3114) and run at 100 V for 100 minutes in a tricine running buffer, followed by transferring to a nitrocellulose membrane for 3 hours at 60 V in a 4 °C cold room. The membrane was blocked in 3 %

BSA solution in tris-buffered saline (TBS) containing 0.1 % Tween-20 (TBS-T buffer) for 1 hour at room temperature, followed by incubation with a primary anti-Aβ antibody (6E10, Covance) overnight at room temperature under constant agitation. The membrane was washed 4 x 15 minutes with TBS buffer and then incubated at room temperature under constant agitation for 2 hours with a horseradish peroxidase conjugated goat anti-mouse secondary antibody in 2 % BSA solution. The membrane was washed with TBS buffer for 4 x 15 minutes, incubated with the Thermo Scientific Supersignal West Pico Chemiluminescent Substrate kit (ThermoScientific #34087) for 5 minutes and visualized with a Fujifilm Luminescent imager. TEM samples were prepared by following a previously reported procedure.[1,21] Briefly, 5 μL aliquots from the native gel electrophoresis experiments were placed onto a sheet of parafilm and incubated for 5 minutes on glow-discharged Formvar/Carbon 300-mesh grids (Electron Microscopy Sciences). Afterward, the grid was stained with syringe-filtered 5% uranyl acetate using 3 × 5 μL drops placed onto parafilm. The grid was placed on the first drop of uranyl acetate and immediately removed, repeated for the second drop, then placed on the third drop to incubate for 1 minute. Excess uranyl acetate was removed using a tissue between drops. Bright field images were obtained using a FEI Osiris operating at 200 kV and 9000X magnification.

**Neuroprotection against $A\beta_{1-42}$-induced toxicity**. Use of brain tissue from therapeutically aborted fetuses (18-20 weeks of gestation) was permitted by the donors after written informed consent. Experiments with human fetal neurons (HFN) were performed in accordance with ethics approval of the Conjoint Health Research Ethics

Board at the University of Calgary. Isolation of HFN was achieved as previously described.[22] Cells were plated at a density of 50 million cells in 25ml media in poly-L-ornithine coated (10μg/ml) T75 flasks in minimal essential medium (MEM) supplemented with 10% fetal bovine serum, 1μM sodium pyruvate, 10μM glutamine, 1X non-essential amino acids, 0.1% dextrose and 1% penicillin/streptomycin (HFN-complete medium; culture supplements from Invitrogen, Burlington, Canada). Medium was supplemented with cytosine arabinoside 25μM (Sigma-Aldrich, St. Louis, MO) to kill dividing astrocytes in order to obtain a neuronal culture of >80% purity. After three cycles with cytosine arabinoside, cells were plated for experiments in poly-L-ornithine coated 96-well plates in a density of 100,000 cells/well in 100μl HFN-complete medium containing cytosine arabinoside. After 2 days, medium was changed to serum-free AIM-V; 1h after, cells were treated with test ligands followed by another hour after with 25μM $A\beta_{1\text{-}42}$. All test and control conditions were performed in quadruplicate wells. Control wells contain media only while the wells used for DMSO contain 10 μM DMSO in media, which was the same final concentration used in the wells that contained Aβ since its stock solution preparation involved DMSO to dissolve the peptide. After 24 h exposure to $A\beta_{1\text{-}42}$, cells were fixed with 4% paraformaldehyde and stored in PBS at 4°C until further processing for immunocytochemistry. For the Cu-$A\beta_{1\text{-}42}$ experiments, cells were pre-treated with ligands for 1 h, followed by a single addition of 1:1 Cu-$A\beta_{1\text{-}42}$ (5, 10, or 25μM). Cu and $A\beta_{1\text{-}42}$ were pre-mixed before addition to the cells. Each experiment was incubated for 24 h, performed in quadruplicate, and then fixed using 4% paraformaldehyde and stored in PBS at 4°C until immunocytochemistry.

**Immunocytochemistry and microscopy**. Fixed cells in each well of 96-well plates were treated with blocking buffer for 1h and then stained with anti-microtubule-associated protein-2 (MAP-2) antibody (dilution 1:1,000; Sigma, Oakville, Canada). After overnight incubation at 4°C, AlexaFluor-546 anti-mouse IgG secondary antibody (dilution 1:250; Invitrogen, Burlington, Canada) was used to visualize MAP-2. Nuclei were stained with Hoechst S769121 (nuclear yellow). Immunofluorescence microscopy was performed using the automated ImageXpress® imaging system (Molecular Devices, Sunnyvale, CA). For each well, 9 specific sites in the center of the well were automatically and digitally sampled through a 10x objective microscope lens, and automated analyses were then performed using the software MetaXpress® with the algorithm "multiwavelength cell scoring" as previously described.[23] The data from the 9 sites/well were averaged to one data point comprising the number of MAP-2 positive cells. Data shown is the mean ± SEM of combined data of n=2 independent experiments performed in quadruplicates (control n=3 experiments), normalized to the number of MAP-2 positive cells of the mean of the respective control wells of each experiment. Statistics was performed using 1-way ANOVA with Dunnett´s multiple comparisons test as post-hoc analysis compared to $A\beta_{1-42}$ and further analyzed using Graphpad prism version 7.

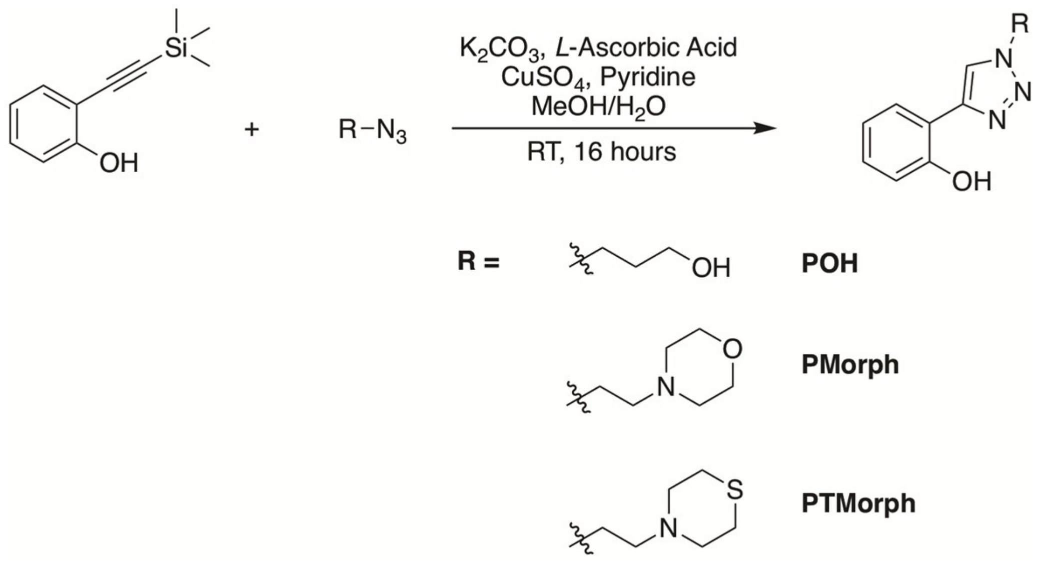


**Scheme S1.** Synthetic routes towards **POH**, **PMorph**, and **PTMorph**.

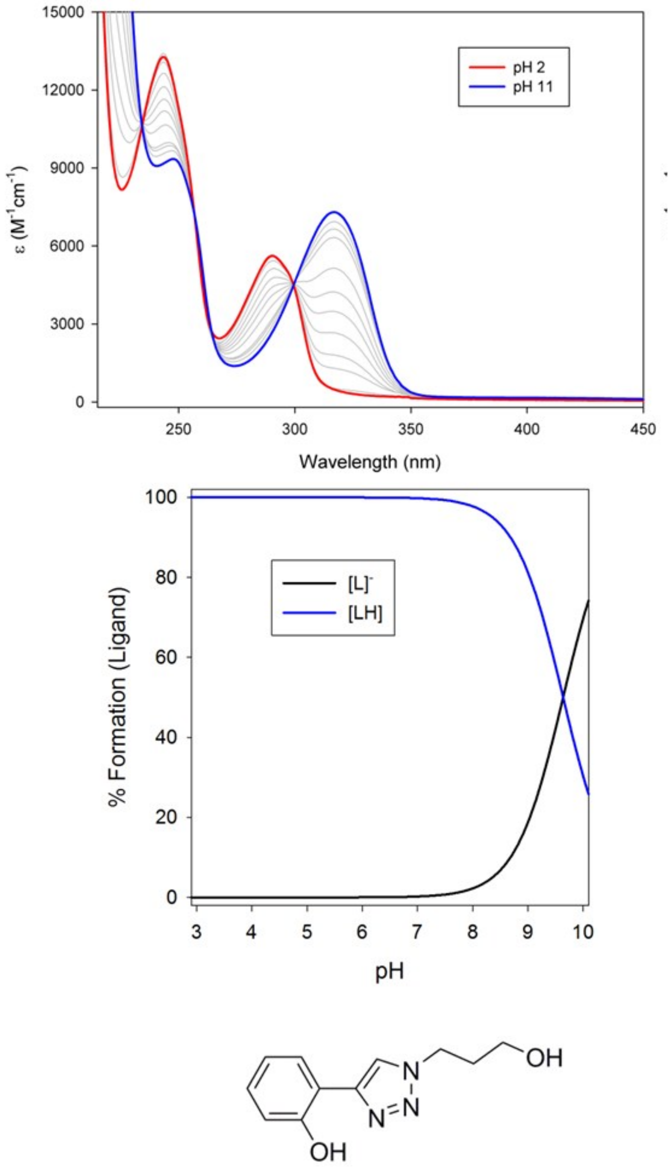


**Figure S1.** (Top) Variable pH UV-vis spectra of **POH** from pH 2 (red) – 11 (blue). (Bottom) Solution speciation diagram of **POH** indicating a predominantly neutral ligand at physiological pH (pH 7.4).

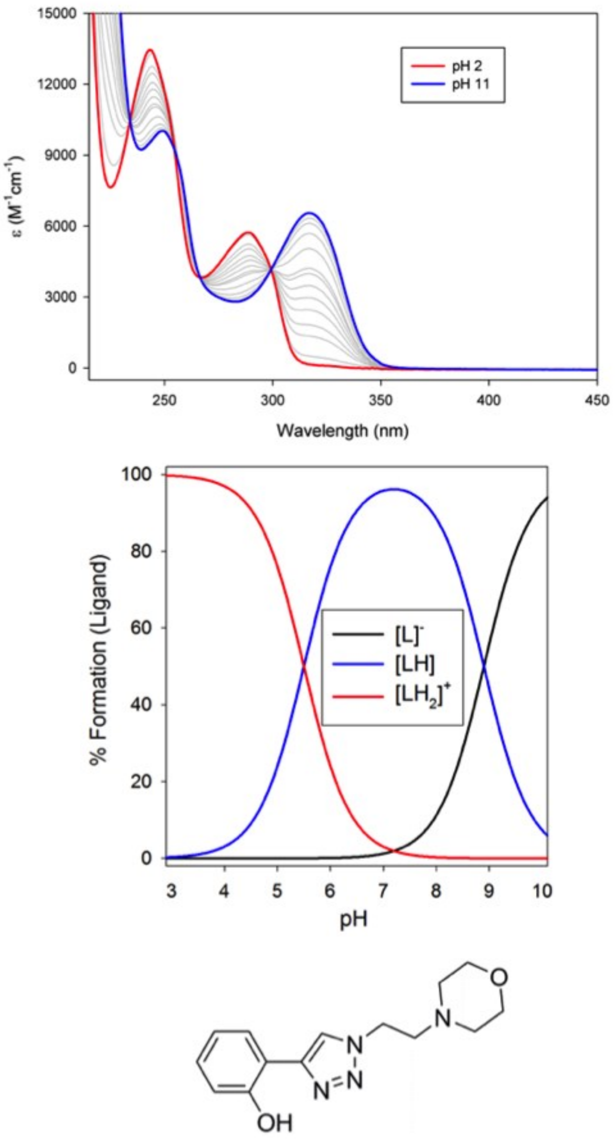


**Figure S2.** (Top) Variable pH UV-vis spectra of **PMorph** from pH 2 (red) – 11 (blue). (Bottom) Solution speciation diagram of **PMorph** indicating a predominantly neutral ligand at physiological pH (pH 7.4).

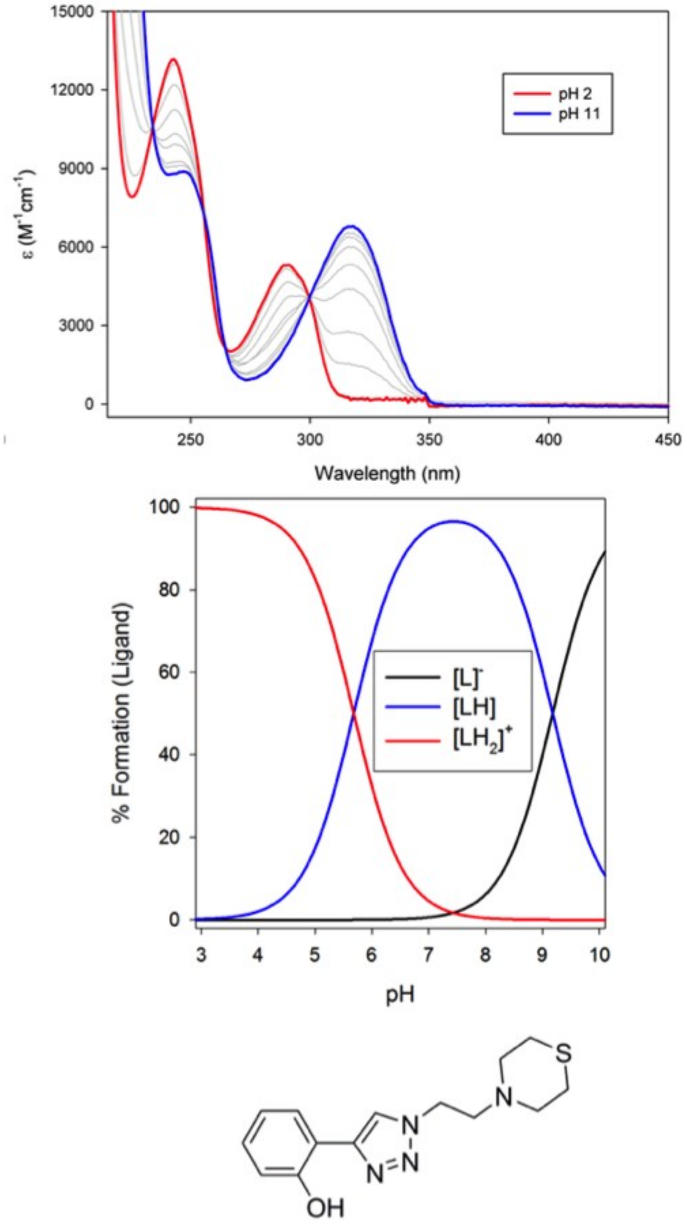


**Figure S3.** (Top) Variable pH UV-vis spectra of **PTMorph** from pH 2 (red) – 11 (blue). (Bottom) Solution speciation diagram of **PTMorph** indicating a predominantly neutral ligand at physiological pH (pH 7.4).

**Table S1.** p$K_a$ values and speciation at physiological pH as determined by variable pH UV-vis and NMR spectroscopy titrations.

| | UV-vis | NMR | |
|---|---|---|---|
| | **p$K_a$ (Phenol)** | **p$K_a$ (R-group)** | **Speciation (pH 7.4)** |
| **POH** | 9.549(6) | n/a | Neutral |
| **PMorph** | 9.559(6) | 5.5(9) | Neutral |
| **PTMorph** | 9.54(1) | 5.6(6) | Neutral |

**Table S2.** Summary of the Lipinski's rules for drug-likeness and determination of the LogBB to predict BBB permeability

| **Calculation**[a] | **POH** | **PMorph** | **PTMorph** | **Optimal Parameters** |
|---|---|---|---|---|
| $M_w$ | 219.244 | 274.324 | 290.392 | ≤ 450 |
| MiLogP | 0.897 | 1.135 | 1.676 | ≤ 5.0 |
| TPSA | 71.174 | 63.418 | 54.184 | ≤ 90 |
| HBA | 5 | 6 | 5 | ≤ 10 |
| HBD | 2 | 1 | 1 | ≤ 5 |
| Lipinski's rules | Pass | Pass | Pass | |
| logBB | -0.78 | -0.63 | -0.41 | > 3.0 (probable); < -1.0 (unlikely) |

[a]$M_w$, molecular weight; MiLogP, calculated logarithm of the octanol-water partition coefficient; TPSA, Topological polar surface area; HBA, Hydrogen bonding acceptor; HBD, Hydrogen bonding donor; logBB, logBB = -0.0148 Å x TPSA + 0.152 Å x MiLogP + 0.139 (logBB > 3.0, readily crosses BBB; log BB < -1.0, low probability of crossing BBB)

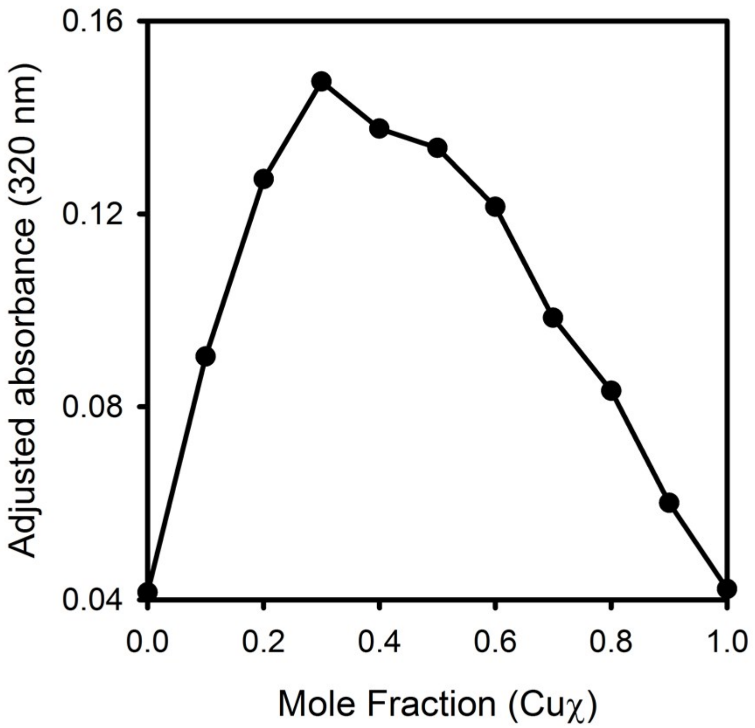


**Figure S4.** Job plot analysis of **PMorph** + $CuCl_2$. Various mole fractions of $CuCl_2$ and **PMorph** were combined in solution (PBS pH 7.4) and their absorbance at 320 nm measured to determine the most favorable stoichiometry between the metal and ligand. The maximum points in the graph are in the range 0.3 – 0.6 mole fraction Cu, suggesting a combination of 2:1 and 1:1 ligand:Cu complexes in solution.

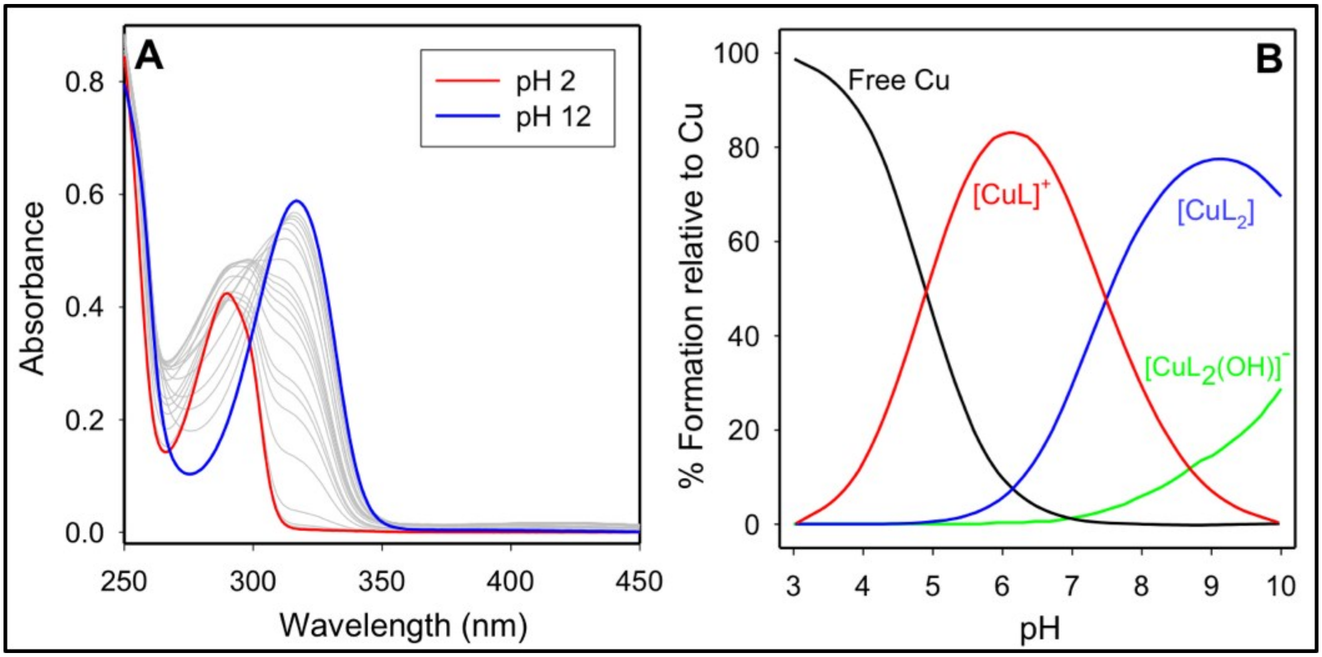


**Figure S5.** **(A)** Variable pH UV-Vis titration of **PMorph** (75 μM) and $CuCl_2$ (37.5 μM) at pH 2 (red spectrum) and pH 12 (blue spectrum). (**B**) Using HypSpec and HySS, the variable pH data was fit to a model including 1:1 (red line) and 2:1 ligand:Cu (blue line) species, free Cu (black line), and a Cu(**PMorph**)$_2$OH (green line) component. At physiological pH 7.4, very little free Cu exists and a combination of 1:1 and 2:1 ligand:Cu species are present.

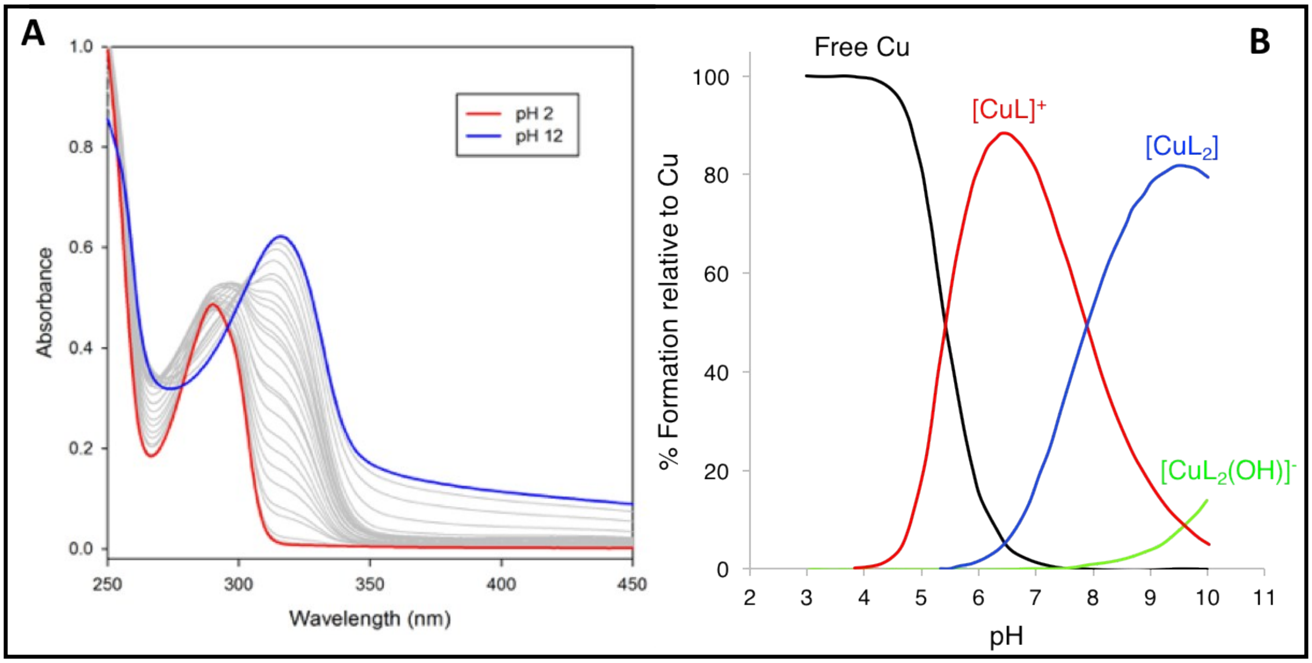


**Figure S6.** (**Top**) Variable pH UV-Vis titration of **PTMorph** (75 μM) and $CuCl_2$ (37.5 μM) where the red spectrum represents pH 2 and the blue spectrum at pH 12. (**Bottom**) Using HypSpec and HySS, the variable pH data was fit to a model incorporating a 1:1 and 2:1 Ligand:Cu ratio along with free Cu and a Cu(**PTMorph**)$_2$OH component. At physiological pH 7.4, a small amount of free Cu exists and a combination of 1:1 and 2:1 Ligand:Cu species are present.

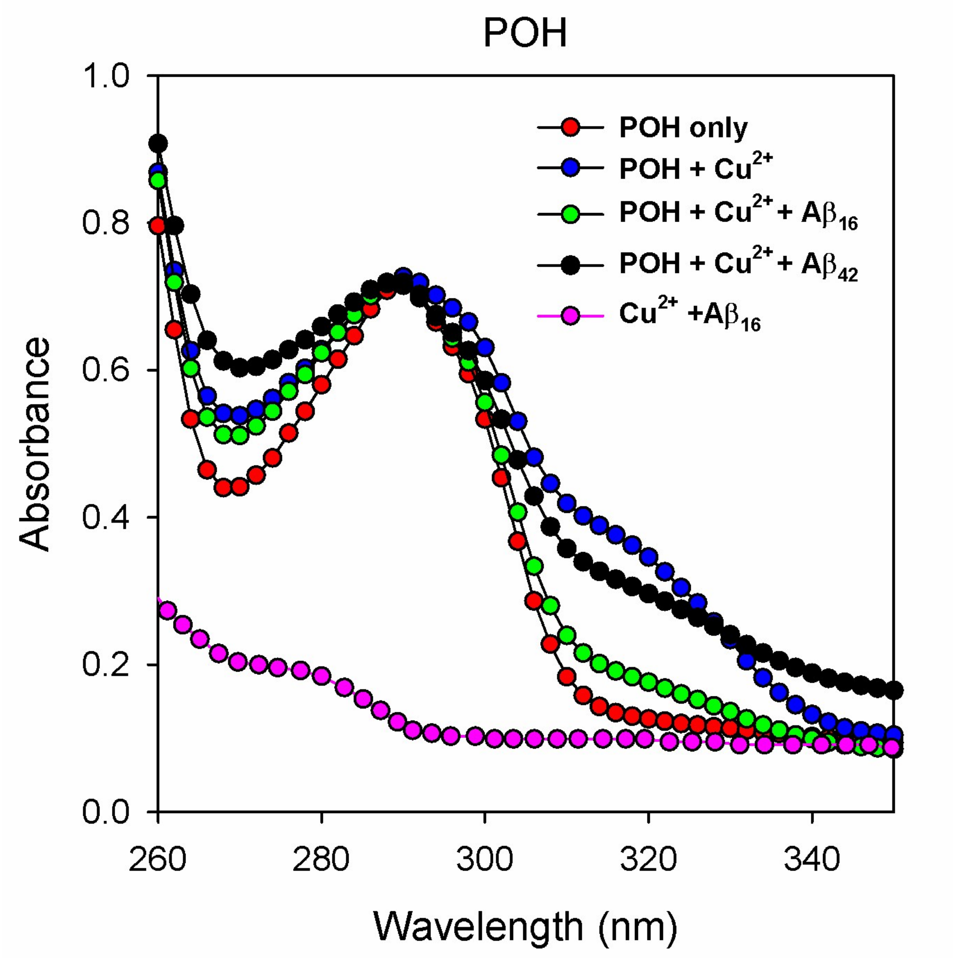


**Figure S7.** Cu competition assay between **POH** and Aβ$_{1\text{-}42/1\text{-}16}$. 75 μM **POH** has a peak maximum at 290 nm, characteristic of a phenolic moiety (Red). Upon addition of 2 eq. **POH** to 1 eq. Cu in the absence of peptide (Blue), a peak at 320 nm is visible, characteristic of Cu complexation. Cu-Aβ$_{1\text{-}42/1\text{-}16}$ were pre-incubated for 20 mins. prior to **POH** exposure to ensure complete Cu-binding to each peptide. Upon addition of 2 eq. **POH** to Cu-Aβ$_{1\text{-}42/1\text{-}16}$, an increase at 320 nm is observed (Black and Green, respectively) indicative of ligand binding to Cu, forming a Cu-**POH** complex. Final Concentrations: 75 μM **POH**, 37.5 μM $CuSO_4$, 45 μM Aβ$_{1\text{-}42/1\text{-}16}$ (1.2 eq.), 50 mM Chelex-treated HEPES buffer pH 7.4.

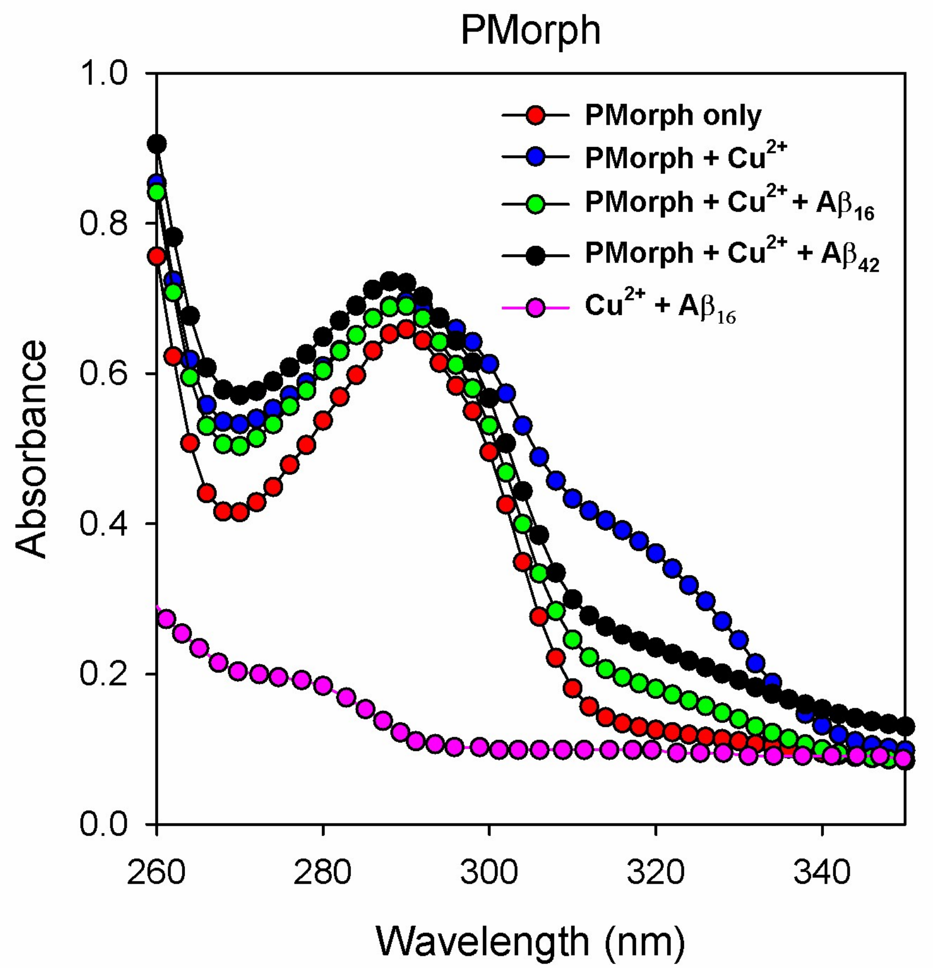


**Figure S8.** Cu competition assay between **PMorph** and $A\beta_{1\text{-}42/1\text{-}16}$. 75 μM **PMorph** has a peak maximum at 290 nm, characteristic of a phenolic moiety (Red). Upon exposure of 2 eq. **PMorph** to 1 eq. Cu in the absence of any peptide (Blue), a peak at 320 nm develops, characteristic of deprotonation of the phenolic moiety to produce a phenolate and binding to Cu. Cu-$A\beta_{1\text{-}42/1\text{-}16}$ were pre-incubated for 20 mins. prior to **PMorph** exposure to ensure complete Cu-binding to each peptide. Upon exposure of 2 eq. **PMorph** to Cu-$A\beta_{1\text{-}42/1\text{-}16}$, a weak peak at 320 nm is observed (Black and Green, respectively) indicating partial de-metallation of Cu from the peptide forming a Cu-**PMorph** complex. Final Concentrations: 75 μM **PMorph**, 37.5 μM $CuSO_4$, 45 μM $A\beta_{1\text{-}42/1\text{-}16}$ (1.2 eq.), 50 mM Chelex-treated HEPES buffer pH 7.4.

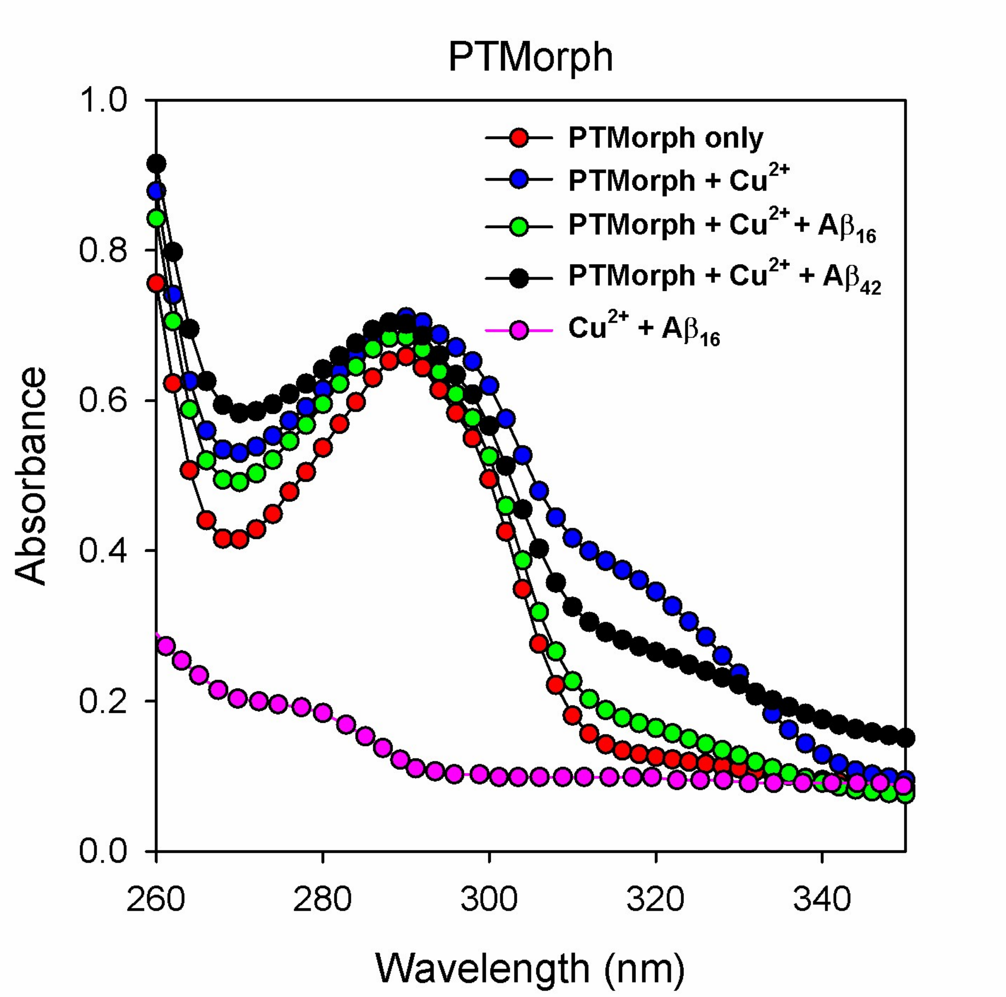


**Figure S9.** Cu competition assay between **PTMorph** and A$\beta_{1\text{-}42/1\text{-}16}$. 75 μM **PTMorph** has a peak maximum at 290 nm, characteristic of a phenolic moiety (Red). Upon exposure of 2 eq. **PTMorph** to 1 eq. Cu in the absence of any peptide (Blue), a peak at 320 nm develops, characteristic of deprotonation of the phenolic moiety to produce a phenolate and binding to Cu. Cu-A$\beta_{1\text{-}42/1\text{-}16}$ were pre-incubated for 20 mins. prior to **PTMorph** exposure to ensure complete Cu-binding to each peptide. Upon exposure of 2 eq. **PTMorph** to Cu-A$\beta_{1\text{-}42/1\text{-}16}$, a weak peak at 320 nm is observed (Black and Green, respectively) indicating partial de-metallation of Cu from the peptide forming a Cu-**PTMorph** complex. Final Concentrations: 75 μM **PTMorph**, 37.5 μM $CuSO_4$, 45 μM A$\beta_{1\text{-}42/1\text{-}16}$ (1.2 eq.), 50 mM Chelex-treated HEPES buffer pH 7.4.

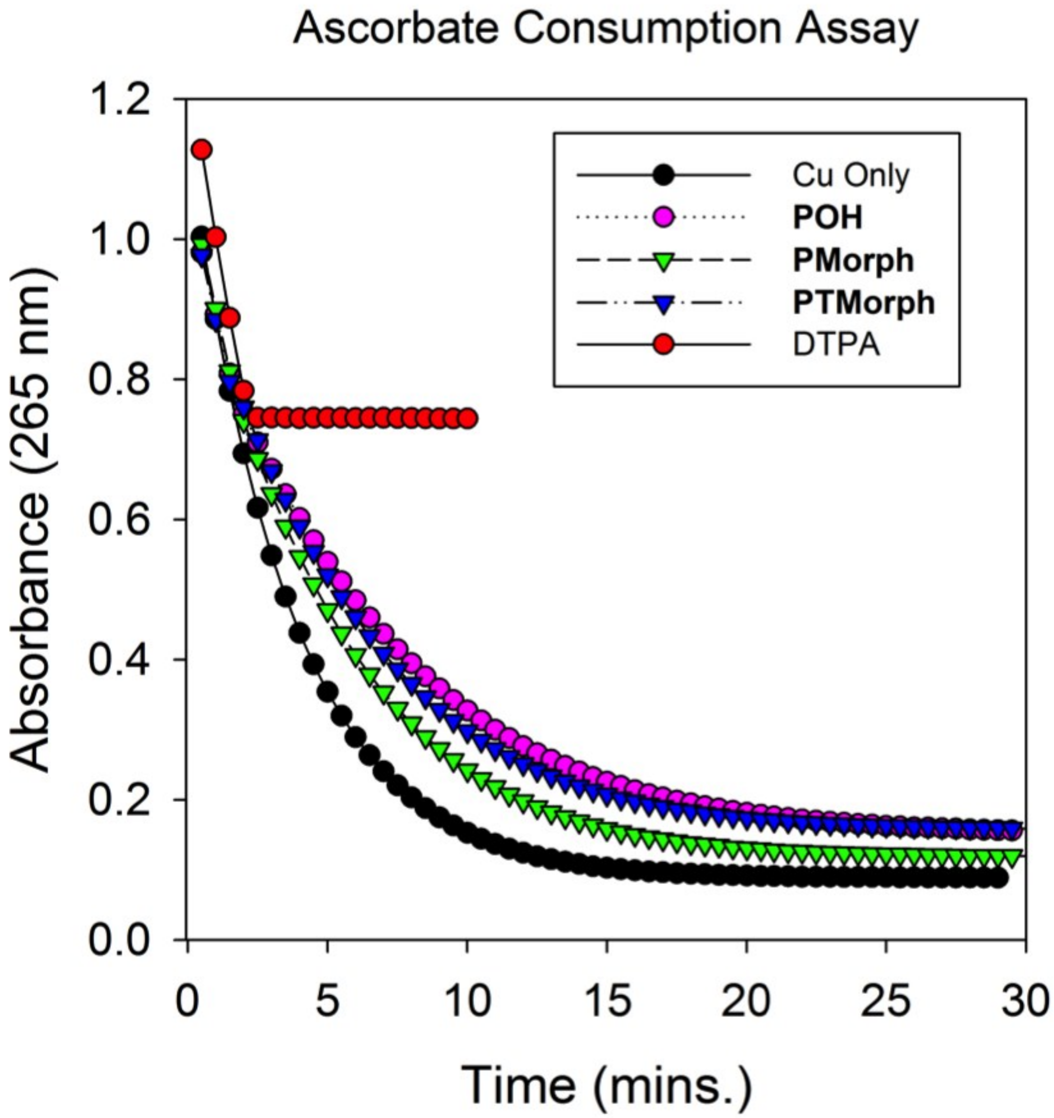


**Figure S10.** Cu-induced ascorbate consumption. When free Cu is in the presence of ascorbate, rapid consumption occurs in 15 mins. post addition of Cu (black). In the presence of 3 eq. DTPA, a known Cu-chelator, rapid cessation of ascorbate consumption occurs upon ligand addition (red). When Cu is in the presence of 10 eq. phenol-triazole ligands, a slight decrease in the rate of ascorbate consumption is observed (pink, green, and blue). Note: ligand addition occurred when absorbance = 0.8.

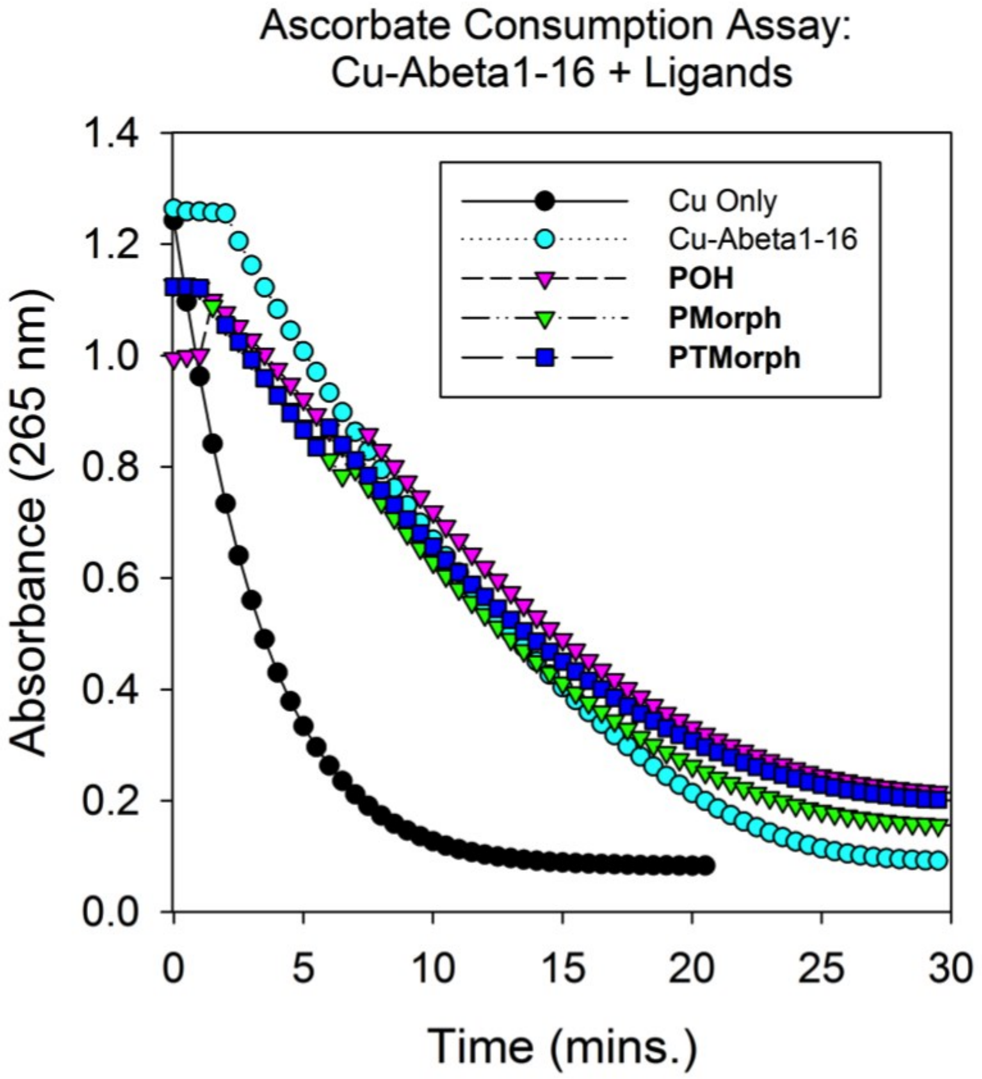


**Figure S11.** Cu-induced ascorbate consumption in the presence of A$\beta_{1\text{-}16}$. When free Cu is in the presence of ascorbate, rapid consumption occurs in 15 mins. post addition of Cu (black). In the presence of 1.2 eq. A$\beta_{1\text{-}16}$, a decrease in the rate of ascorbate consumption occurs, but plateaus around 25 mins. post Cu addition (turquoise). When Cu-A$\beta_{1\text{-}16}$ is in the presence of 10 eq. phenol-triazole ligands, negligible changes in the rate of ascorbate consumption is observed (pink, green, and blue).

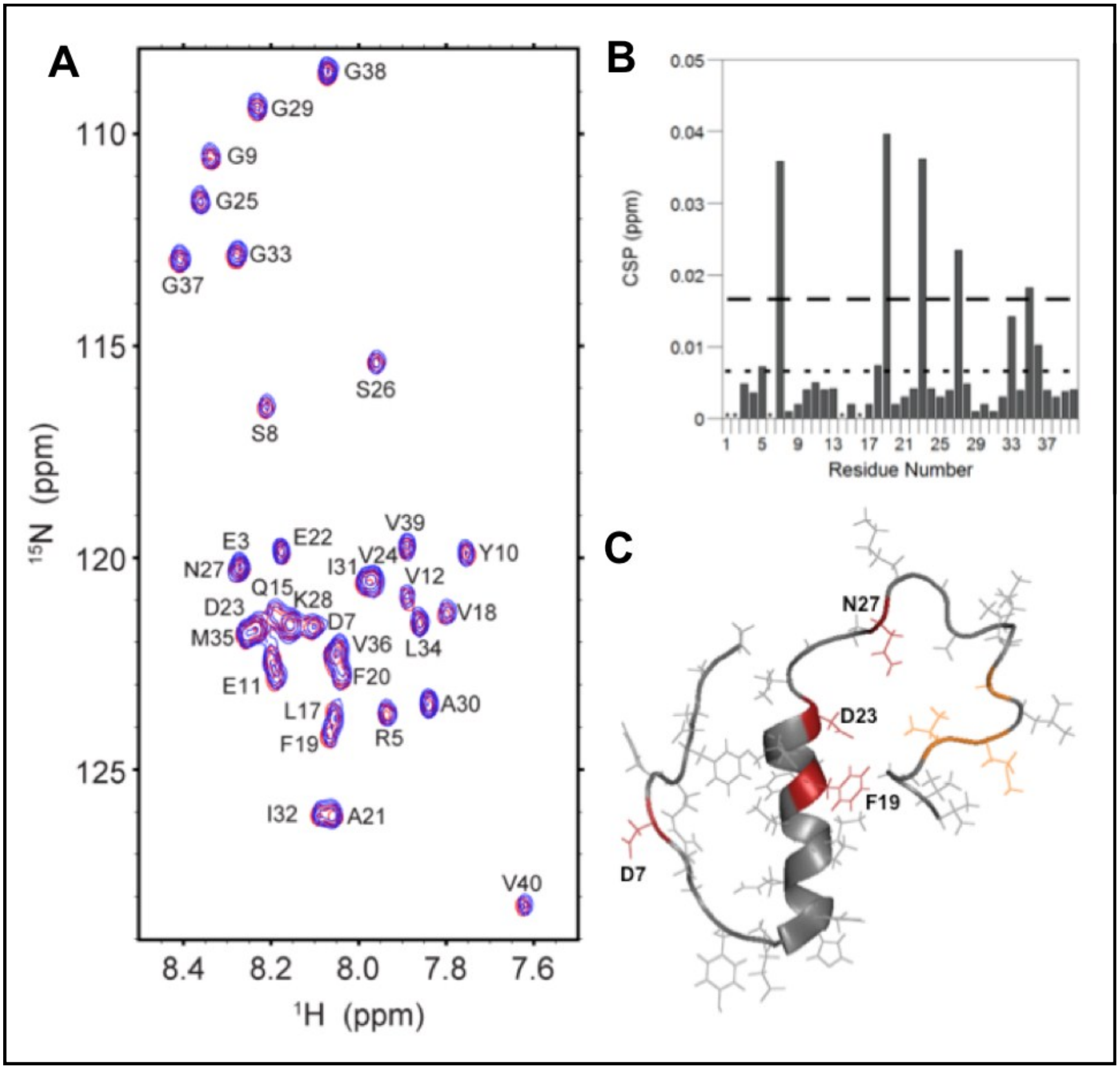


**Figure S12.** 2-D $^{1}H$-$^{15}N$ SOFAST NMR experiments using $^{15}N$-labeled A$\beta_{1\text{-}40}$ and 0-10 eq. **PMorph**. (**A**) 2-D $^{1}H$-$^{15}N$ SOFAST NMR spectrum showing the assignment of specific amino acid residues in the $^{15}N$-labeled A$\beta_{1\text{-}40}$ peptide. (**B**) Summary of the specific amino acid residues that have shifted at 10 eq. **PMorph**. The dotted line represents the average CSP while the dashed line is the average + one standard deviation, which was used to identify which CSP were statistically relevant. (**C**) A$\beta_{1\text{-}40}$ solution NMR structure (PDB 2LFM)[60] highlighting specific amino acid residues that have significant CSP shifts (Red, > 0.02 ppm shift) and moderate CSP shifts (Orange, between 0.01 and 0.02 ppm).

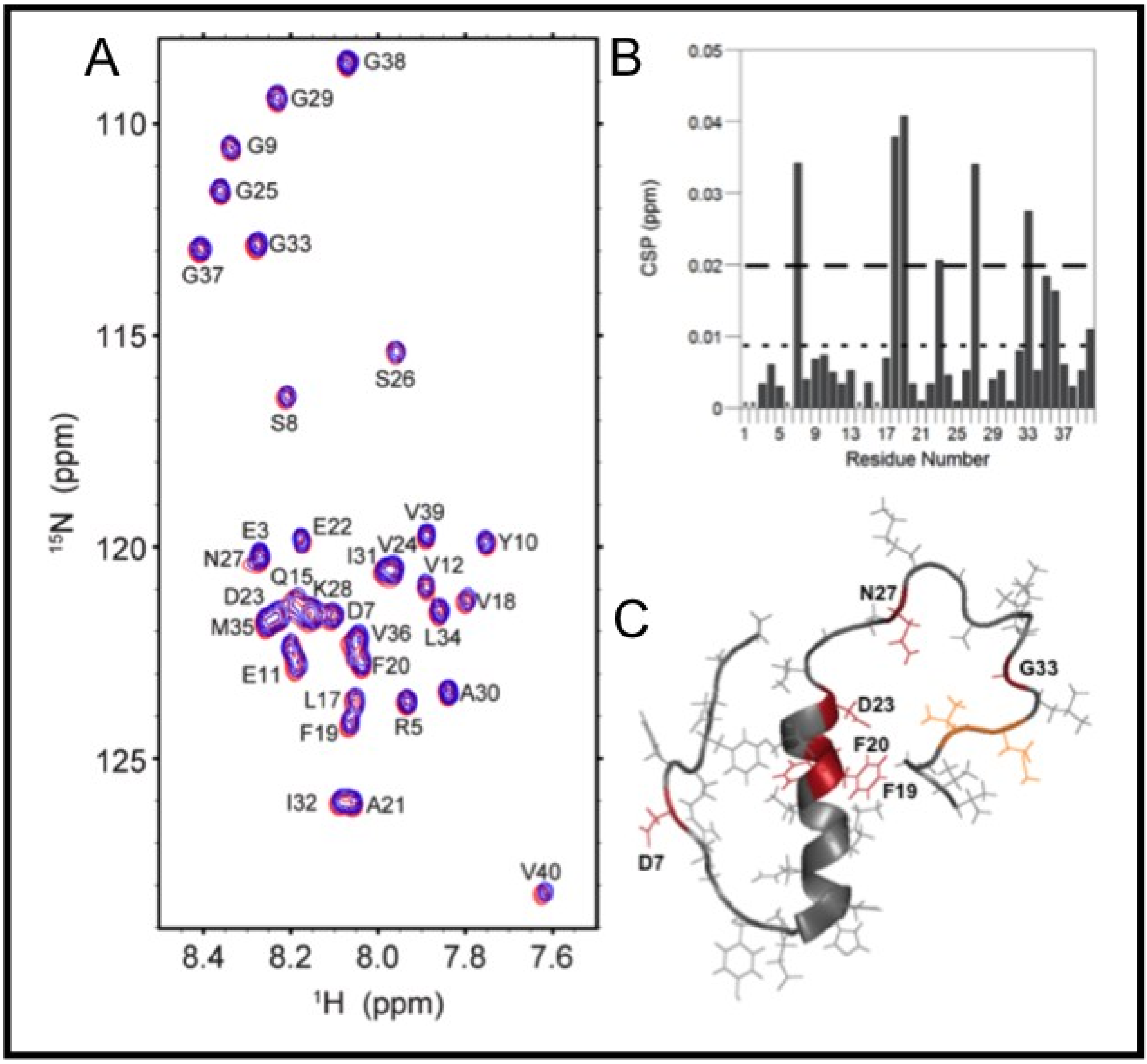


**Figure S13.** 2-D $^1$H-$^{15}$N SOFAST NMR experiments using $^{15}$N-labeled A$\beta_{1\text{-}40}$ and 0-10 eq. **PTMorph**. (**A**) 2-D $^1$H-$^{15}$N SOFAST NMR spectra showing the assignment of specific amino acid residues in the A$\beta_{1\text{-}40}$ peptide. (**B**) Summary of the specific amino acid residues that have shifted at 10 eq. **PTMorph**. The dotted line represents the average CSP while the dashed line is the average + one standard deviation. (**C**) A$\beta_{1\text{-}40}$ solution NMR structure highlighting specific amino acid residues that have significant CSP shifts (Red, > 0.03 ppm shift) and moderate CSP shifts (Orange, between 0.015 and 0.03 ppm).

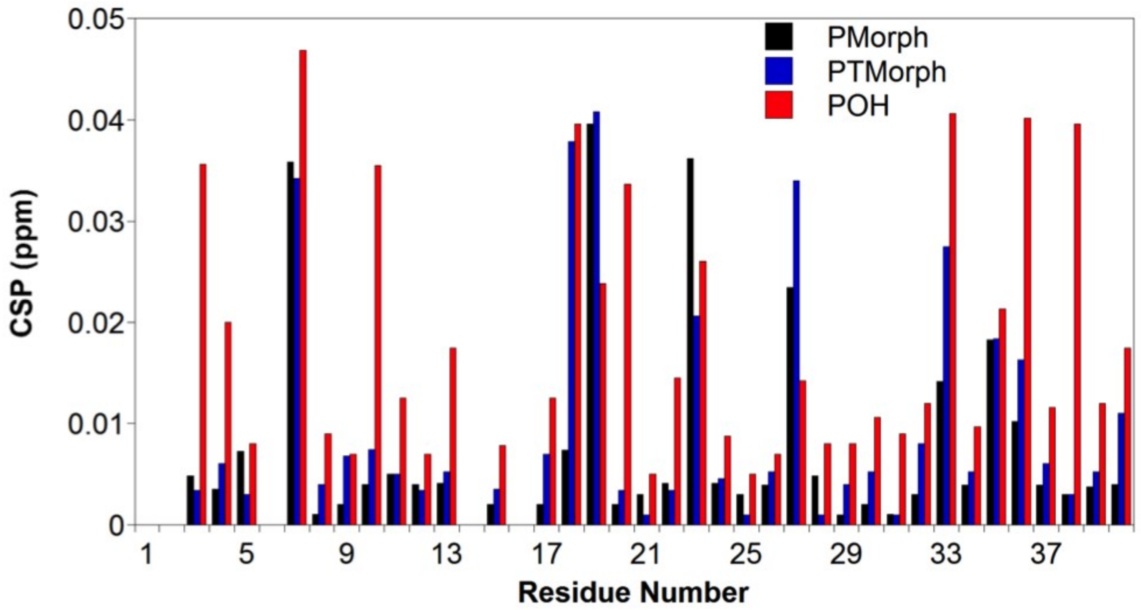


**Figure S14**. Summary of the 2D $^{1}H$-$^{15}N$ SOFAST NMR experiment for the phenol-triazole series of ligands, demonstrating which amino acid residues have CSP's associated with each ligand. D7, F19, D23, G33, M35, and V36 were found to have statistically relevant CSP's across all three investigated ligands.

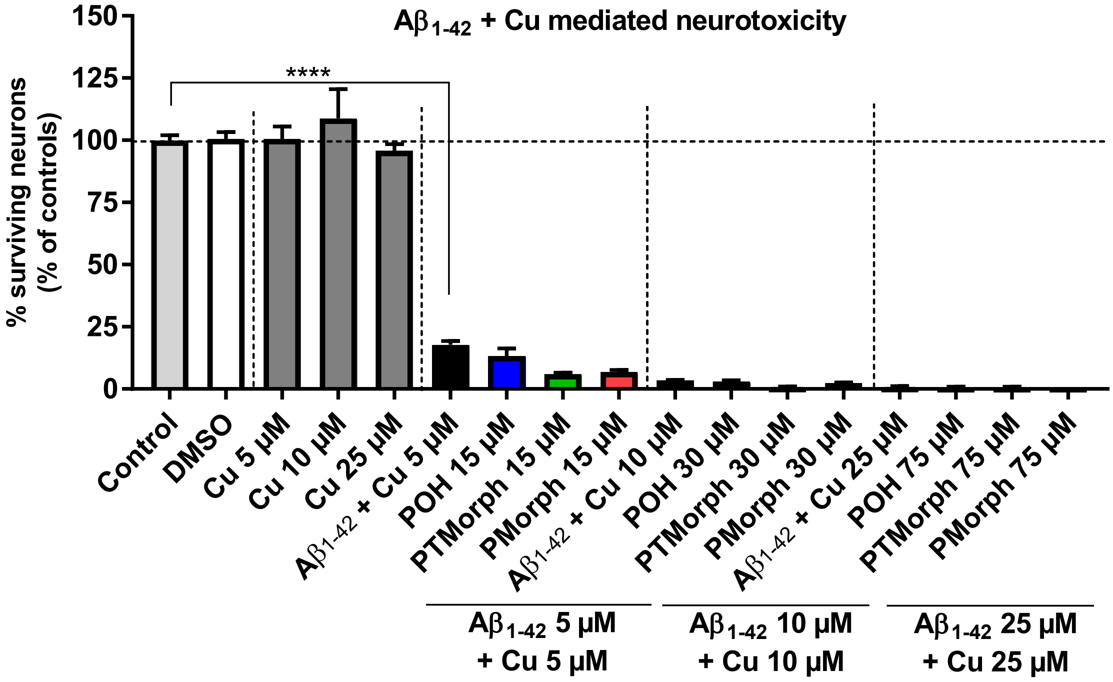


**Figure S15**. Cu-A$\beta_{1\text{-}42}$ neurotoxicity assay using human fetal neurons (HFN). Variable concentrations of Cu were added to wells containing HFN and was well tolerated up to 25 μM. When 5, 10, or 25 μM Cu-A$\beta_{1\text{-}42}$ were present with HFN, significant neuronal death was observed. When 3 eq. ligand was added at each Cu-A$\beta_{1\text{-}42}$ concentration, no neuroprotection was observed under these experimental conditions.

## References


1. M. R. Jones, E. L. Service, J. R. Thompson, M. C. P. Wang, I. J. Kimsey, A. S. DeToma, A. Ramamoorthy, M. H. Lim and T. Storr, *Metallomics*, 2012, **4**, 910-920.
2. M. R. Jones, C. Dyrager, M. Hoarau, K. J. Korshavn, M. H. Lim, A. Ramamoorthy and T. Storr, *J. Inorg. Biochem.*, 2016, **158**, 131-138.
3. V. Hong, S. I. Presolski, C. Ma and M. G. Finn, *Angew. Chem. Int. Ed.*, 2009, **48**, 9879-9883.
4. A. Arcadi, S. Cacchi, M. Del Rosario, G. Fabrizi and F. Marinelli, *J. Org. Chem.*, 1996, **61**, 9280-9288.
5. P. Leeson, *Nature*, 2012, **481**, 455-456.
6. D. E. Clark and S. D. Pickett, *Drug Discov. Today*, 2000, **5**, 49-58.
7. P. S. Gans, A.; Vacca, A., *Ann. Chim.*, 1999, **89**, 45-49.
8. L. Alderighi, P. Gans, A. Ienco, D. Peters, A. Sabatini and A. Vacca, *Coord. Chem. Rev.*, 1999, **184**, 311-318.
9. Y. Manevich, K. D. Held and J. E. Biaglow, *Radiat. Res.*, 1997, **148**, 580-591.
10. K. M. Lincoln, T. E. Richardson, L. Rutter, P. Gonzalez, J. W. Simpkins and K. N. Green, *ACS Chem. Neurosci.*, 2012, **3**, 919-927.
11. R. Re, N. Pellegrini, A. Proteggente, A. Pannala, M. Yang and C. Rice-Evans, *Free Radic. Biol. Med.*, 1999, **26**, 1231-1237.
12. S. Vivekanandan, J. R. Brender, S. Y. Lee and A. Ramamoorthy, *Biochem. Biophys. Res. Commun.*, 2011, **411**, 312-316.
13. S. I. Yoo, M. Yang, J. R. Brender, V. Subramanian, K. Sun, N. E. Joo, S. H. Jeong, A. Ramamoorthy and N. A. Kotov, *Angew. Chem. Int. Ed.*, 2011, **50**, 5110-5115.
14. N. L. Fawzi, J. Ying, D. A. Torchia and G. M. Clore, *J. Am. Chem. Soc.*, 2010, **132**, 9948-9951.
15. v. Maestro, Schrodinger, LLC, New York, NY, 2014.
16. G. M. Sastry, M. Adzhigirey, T. Day, R. Annabhimoju and W. Sherman, *J. Comput. Aided Mol. Des.*, 2013, **27**, 221-234.
17. J. Shelley, A. Cholleti, L. Frye, J. Greenwood, M. Timlin and M. Uchimaya, *J. Comput. Aided Mol. Des.*, 2007, **21**, 681-691.
18. W. Sherman, H. S. Beard and R. Farid, *Chem. Biol. Drug Des.*, 2006, **67**, 83-84.
19. R. A. Friesner, J. L. Banks, R. B. Murphy, T. A. Halgren, J. J. Klicic, D. T. Mainz, M. P. Repasky, E. H. Knoll, M. Shelley, J. K. Perry, D. E. Shaw, P. Francis and P. S. Shenkin, *J. Med. Chem.*, 2004, **47**, 1739-1749.
20. T. A. Halgren, R. B. Murphy, R. A. Friesner, H. S. Beard, L. L. Frye, W. T. Pollard and J. L. Banks, *J. Med. Chem.*, 2004, **47**, 1750-1759.
21. L. M. F. Gomes, R. P. Vieira, M. R. Jones, M. C. P. Wang, C. Dyrager, E. M. Souza-Fagundes, J. G. Da Silva, T. Storr and H. Beraldo, *J. Inorg. Biochem.*, 2014, **139**, 106-116.
22. G. G. Vecil, P. H. Larsen, S. M. Corley, L. M. Herx, A. Besson, C. G. Goodyer and V. W. Yong, *J. Neurosci. Res.*, 2000, **61**, 212-224.
23. R. C. Cua, L. W. Lau, M. B. Keough, R. Midha, S. S. Apte and V. W. Yong, *Glia*, 2013, **61**, 972-984.
